# Heat transfer analysis of the mixed convective flow of magnetohydrodynamic hybrid nanofluid past a stretching sheet with velocity and thermal slip conditions

**DOI:** 10.1371/journal.pone.0260854

**Published:** 2021-12-14

**Authors:** Muhammad Ramzan, Abdullah Dawar, Anwar Saeed, Poom Kumam, Wiboonsak Watthayu, Wiyada Kumam

**Affiliations:** 1 Department of Mathematics, KMUTT Fixed Point Research Laboratory, Room SCL 802 Fixed Point Laboratory, Science Laboratory Building, Faculty of Science, King Mongkut’s University of Technology Thonburi (KMUTT), Bangkok, Thailand; 2 Center of Excellence in Theoretical and Computational Science (TaCS-CoE), Science Laboratory Building, Faculty of Science, King Mongkut’s University of Technology Thonburi (KMUTT), Thung Khru, Bangkok, Thailand; 3 Department of Mathematics, Abdul Wali Khan University, Mardan, Khyber Pakhtunkhwa, Pakistan; 4 Department of Medical Research, China Medical University Hospital, China Medical University, Taichung, Taiwan; 5 Department of Mathematics and Computer Science, Applied Mathematics for Science and Engineering Research Unit (AMSERU), Program in Applied Statistics, Faculty of Science and Technology, Rajamangala University of Technology Thanyaburi, Thanyaburi, Pathumthani, Thailand; Central University of Karnataka, INDIA

## Abstract

The present study is related to the analytical investigation of the magnetohydrodynamic flow of *Ag* − *MgO*/ water hybrid nanoliquid with slip conditions via an extending surface. The thermal radiation and Joule heating effects are incorporated within the existing hybrid nanofluid model. The system of higher-order partial differential equations is converted to the nonlinear system of ordinary differential equations by interpreting the similarity transformations. With the implementation of a strong analytical method called HAM, the solution of resulting higher-order ordinary differential equations is obtained. The results of the skin friction coefficient, Nusselt number, velocity profile, and temperature profile of the hybrid nanofluid for varying different flow parameters are attained in the form of graphs and tables. Some important outcomes showed that the Nusselt number and skin friction are increased with the enhancement in Eckert number, stretching parameter, heat generation parameter and radiation parameter for both slip and no-slip conditions. The thermal profile of the hybrid nanofluid is higher for suction effect but lower for Eckert number, stretching parameter, magnetic field, heat generation and radiation parameter. For both slip and no-slip conditions, the hybrid nanofluid velocity shows an upward trend for both the stretching and mixed convection parameters.

## 1. Introduction

In recent decades, scientists and researchers have focused their attention on the study of a new class of fluid known as a hybrid nanofluid. Fluids with a higher thermal conductivity than nanofluids and base fluid are known as hybrid nanofluids. Hybrid nanofluids are formed by diffusing two sorts of nanoparticles in the carrier liquid. Mechanically to improve the transmission of heat in a variety of engineering and manufacturing processes the hybrid nanoparticles energy transition has been devolved. The hybrid nanofluid has strong thermal reliability as compared to the classical and base liquid. The numerous applications of hybrid nanofluid in industries and engineering mechanisms are heat pumps, heat capacitors microelectronics, digital cooling, car radiators, nuclear power stations, coolant in writing and machine tools, chemical producers, nano-drag shipments, automobile power creation, cancer treatment, and so forth. Several studies on hybrid nanofluid being conducted by many researchers and scientists as a result of their significant applications. Shoaib et al. [1] considered the spinning flow of nanofluid flow with radiation effects inside the stretching surface and noticed a variation of rotation parameter for velocity profile. Hanaya et al. [2] explained the micropolar hybrid nanofluid flow toward the curved stretching surface containing SWCNT and MWCNT nanoparticles and applied the bvp4c-shooting scheme for the examination of their model results. Liaquat et al. [3] numerically computed the MHD hybrid nanofluid flow toward the shrinking surface for stability analysis and dual solutions and their problem consists of *C*_*u*_ − *Al*_2_*O*_3_ − water the base fluid. They found that for increasing values of suction and radiation parameters temperature is enhanced (decrease) for both solutions. Shoaib et al. [4] scrutinized the phenomena of viscous dissipation in 3D MHD hybrid nanofluid flow via rotating disk. In this research work, it is noted that magnetic field reduced both the radial and azimuthal skin frictions coefficients. Ahmad et al. [5] pointed out the study of *Go* + Silver(*Ag*) in Maxwell hybrid nanoliquid for the improvement of thermal performance. Their concluding remarks illustrated that the heat transition is improved through the addition of silver volume fraction with graphene oxide. Alhussain et al. [6] inspected the influence of variable viscosity in a blood-based two-dimensional Casson hybrid nanofluid through the stretching sheet by introducing a magnetic field perpendicular in a flow field. From this analysis, the authors have demonstrated that with the increment of nanomaterials concentration in the base fluid, the thermal expansion rate is increased but the specific heat capacity decreased. Sreedevi et al. [7] perceived a numerical study for gyrotactic microorganism’s past over a swirling cylinder and examined that microorganism density is raised for Weissenberg number. In addition, Roy et al. [8] inspected the second-grade nanoliquid flow across an extending surface along with heat propagation. They reported that the heat flux is condensed but wall shear stress is improved through the variation of velocity ratio parameter.

The magnetohydrodynamic is in the domain of physical science that is concerned with the magnetic and electrically conducting fluids properties including saltwater, liquid metals, electrolytes, and plasma. The magnetohydrodynamic is engaged in a lot of engineering and industrial applications such as generators and electronic filters, heat exchangers, power pumps, cooling of the reactors, power generation, plasma, MHD sensor, magnetic drugs targeting, crystal intensification, and so forth. Many researchers and scientists are interested to analyze the behavior due to the influence of the magnetic field, notably in the context of boundary layer flow problems. Tlili et al. [9] scrutinized the MHD flow of hybrid ferrofluid with asymmetrical heat transfer rise and fall effect. They studied the impact of Fe_3_*O*_4_(magnetic oxide) and CoFe_2_*O*_4_ (cobalt iron oxide) ferrous particles embedded in an *H*_2_*O* −*EG* (ethylene) (50–50%) mixture in their hybrid fluid model. Kempannagrari et al. [10] contemplated a problem to explain the MHD non-Newtonian fluid flow above the exponentially stretching surface along with joule heating effects. From this study, it is concluded that the temperature of the fluid is escalated with the escalation of the heat source parameter and Eckert number. Kumar et al. [11] considered the influence of Cattaneo-Christove heat flux on the problem of MHD electrically conducting fluid past a wedge and a cone. They made a comparison of momentum boundary layer thickness over the wedge and cone and found that the momentum boundary layer thicknesses are higher for flow on the wedge as compared to the cone. Ramudu et al. [12] presented a model of MHD Casson nanofluid flow under the stretching surface to check the effect of Brownian and thermophoresis behavior and obtained that the Lewis flow parameter decreased the concentration of the nanofluid. Kumar et al. [13] explored the characteristics of first and second-order velocity slip conditions on the MHD flow of micropolar nanofluid through the stretching surface and their result shows that second-order velocity slip conditions declined the temperature of the nanofluid but enhanced the velocity of the nanofluid. Ramdevi et al. [14] reported the solution of three-dimensional Carreau fluid flow on the bidirectional stretching sheet along with modified Fourier law and chemical reaction impact. Kumar et al. [15] analyzed the modeling of MHD flow of micropolar fluid toward the slandering stretching surface in the attendance of viscous dissipation. From this investigation, it is observed that the couple stress coefficient is higher for the microrotation parameter. Idowu and Falodun [16] presented the heat-mass transfer behavior in the magnetohydrodynamic non-Newtonian Casson nanofluid flow on the plate. For acquiring the solution of the problem, their converted ODEs were solved with the implementation of the spectral HAM. Islam et al. [17] discussed the MHD nano liquid flow through graphene oxide (*Go*) and copper (*Cu*) hybrid nanofluid. Their outcomes indicated that the Reynolds number declined the fluid temperature profile. Jain and Bhargava [18] scrutinized the MHD free convective non-Newtonian fluid flow inside a wavy enclosure with the use of the Meshfree method with the implementation of the magnetic field in the horizontal direction in which they noted that transfer of heat is much higher when Rayleigh number is high. El-Dabe et al. [19] used a numerical scheme for the study of MHD non-Newtonian power-law nanofluid flow in a non-Darcy porous media toward the inclined channel with Ohmic dissipation. In this evaluation, they found that nanoparticles concentration is the increasing function of the Eckert number. Kotha et al. [20] discussed the presence of the gyrotactic microorganism in two-dimensional MHD water-based nanofluid flow through the vertical plate and explained that the bioconvection Peclet number boosted the local Sherwood number impact. Waqas et al. [21] proposed the mathematical modeling of electrically conducting Oldroyd-B nanoliquid flow surrounded by a permeable medium across the rotating frame. Aziz and Shams [22] made the estimation of heat transmission in MHD non-Newtonian nanoliquid flow under the stretching sheet. They showed that the porosity parameter shows decrement in entropy generation, but the inverse trend of Brinkman number is observed. Gupta et al. [23] introduced the two-dimensional Williamson nanoliquid model with the variable thickness and also depicted the effects of different emerging parameters over fluid velocity, temperature concentration.

The improvements in fluid heat transfer rate have attracted a lot of attention from scientists and engineers because of their extensive and practical uses in medical, industrial, and technical domains. The heat transmission through the hybrid nanofluid has a broad spectrum of uses in serval mechanical and engineering science, chemical analysis and biological sectors such as food processing insulating materials, polymers solar energy and reactor fluidizations, cooling of microchips, heat exchangers, heat pipes, solar water heating, extraction of geothermal power, glass manufacturing, hydraulic breaks and so on. Several researchers have analyzed the effect of heat transition on various flow conditions as a result of these varied uses. AshwinKumar et al. [24] presented the numerical solution of two-dimensional MHD hybrid nanofluid flow with heat transfer effect toward the different geometries by using the Runge-Kutta method. In their investigation, they take water as a base fluid and the nanoparticles are CuO and *Al*_2_*O*_3_. Samrat et al. [25] introduced the mathematical modeling of magnetohydrodynamic dusty-nano and dusty hybrid-nano liquid with the occurrence of heat transfer behavior. They found that higher heat transfer occurs in dusty hybrid-nano liquid as compared to the dusty nano liquid. Chalavadi et al. [26] considered the study of MHD Sakiadis flow of Casson/Carreau hybrid nanofluid flow over the moving needle in the presence of heat transfer in which they discussed that with the enhancement of nanoparticles volume fraction the temperature distribution of Carreau hybrid fluid model is augmented than the Casson fluid model. Ashwin Kumar et al. [27] adopted the regular perturbation technique for the investigation of the heat transfer effect over the unsteady MHD flow of *Al*_50_*Cu*50 / *Ag* − water nanofluid through the elongate surface. Mabood et al. [28] studied the MHD flow of an unsteady hybrid nanofluid past a slandering surface along with the influence of heat transfer impact. They noted that the skin friction coefficient of the hybrid nano-liquid is reduced when the magnetic field parameter is increased. Mabood et al. [29] described the impacts of heat transfer and nonlinear radiation on the three-dimensional unsteady MHD flow of *Fe*_3_*O*_4_/graphene-water hybrid nanofluid. They observed that the enlargement in heat transfer is more favorable in hybrid nanofluid as compared to the usual nanofluid. Tlili et al. [30] deliberated the theoretical and experimental study of three-dimensional MHD flow of *AA*7072−*AA*7075/methanol hybrid nanofluid on the uneven thickness surface with heat transfer effects. They noticed that the Nusselt number of hybrids nanofluid is raised for wall thickness parameter. Akbari et al. [31] explained the heat propagation in a non-Newtonian hydrodynamic nanoliquid flow containing the volume fraction 0.5%-1.5% of the solid nanoparticles Alumina. They figured out that betterment in heat transfer is occur because of the enhancement in the quantity of nanoparticulate. Xiong et al. [32] conducted the experimental analysis of the power-law in *Cu* − water nanoliquid model by using energy transmission and flow nature and used finite volume method for the description of the problem and found that the ratio between Nusselt number and skin friction becomes high up to 3.57% and 0.08% when the Grahsof and Reynolds number increases. Mahabaleshwar et al. [33] designed a model of MHD mixed convective-radiative flow in a permeable medium toward the accelerating surface. Their findings from this study show that the boundary layer thickness is reduced for all the involving functions because of the increment of the Richardson parameter. Mukthar et al. [34] initiated the influence of energy transition and thermal radiation in a flow of MHD Maxwell Copper-water *Cu* − *H*_2_*O* and Molybdenumdisuflide MoS_2_ − *H*_2_*O* nanofluid past a stretched flat plate. They experimented that, *Cu* − *H*_2_*O* based nanoliquid has higher thermal efficiency as compared to the MoS_2_ − *H*_2_O nanofluid. Hashim et al. [35] introduced the mass and energy transmission characteristics in 2D time-dependent Williamson nanoliquid flow via heated sheet and concluded that the thermophoresis parameter improved the fluid temperature. Hayat et al. [36] discoursed the MHD flow of unsteady Jeffrey nanofluid through the stretching cylinder. From this observation, we can analyze that fluid viscosity is intensifying with the increasing of Schmidt number *Sc*. Khan et al. [37] investigated the energy diffusion processes in Walter-B ferrofluid using gyrotactic microorganisms in free convective nanocomposites slip flow. In this paper, they observed that the bioconvection Peclet and Lewis number declined the motile density profile. In another investigation of heat transfer, Waini et al. [38] reported the hybrid MHD nanofluid flow over the permeable stretching wedge by considering the heat transition and magnetic effects and also explained the comparison of heat transfer between hybrid nanoliquid and regular nanofluid and studied that hybrid nano-fluid increases more heat transfer rate as compared to regular nanofluid.

Joule heating effects occur when an electric current passes through a conductor and produces heat. The use of the Joule heating effect is in multiple devices and industrial processes. The food processing, scanning electron microscope, infrared-thermal images, electric radiative space heater, incandescent light bulb, laboratory water bath, electric tabletop hotplate, clothes iron, soldering iron, fan heater, hairdryer and cartridge heater are some important applications of the Joule heating effects. The researchers and engineers discussed the Joule heating effect in their research area in light of the above applications of the Joule heating effect. Alaidrous et al. [39] explored the 3D magnetic flow of a hyperbolic tangent nanofluid in porous media toward the extending surface and obtained that fluid temperature is increased for joule heating parameter. Hafeez et al. [40] studied the non-Newtonian Oldroyed-B nanofluid flow along with the characteristics of Cattaneo-Christove heat flux theory. In this work, they disclosed that the concentration of the Oldroyed-B nanofluid is raised for the thermophoresis parameter. Khan et al. [41] carried out the numerical study of hydromagnetic three-dimensional Casson nanofluid flow via rotating cylinder along with entropy and Joule heating effects and found that magnetic field parameter upsurges the nanofluid velocity profile. Kumar et al. [42] employed the shooting method along with the RK4 scheme to study the influence of the Joule heating effect over the flow of Williamson nanofluid toward the stretching sheet. In this analysis, they noted that the entropy of the system is heightened with the improvement of the Brinkman parameter. Khashi’ie et al. [43] studied the occurrence of Joule heating and heat transmission effects in a hybrid nanofluid flow through the shrinking cylinder. Further, they take *Cu* − *Al*_2_*O*_3_ nanoparticles and water as base fluid in their present investigation. Uddin et al. [44] discovered the different aspects of the Joule heating effect on the mixed convection MHD flow. From their conclusion, we conclude that the material magnetic parameter boosted the skin friction coefficient. Kazemi et al. [45] provided an analytical solution of the fourth-grade nanofluid flow along the duct walls with the existence of viscous dissipation and Joule heating impacts. Their results revealed that heat transfer over the duct walls shows decrement up to 40% when the Hartman number is upgraded. Rasheed et al. [46] addressed the MHD boundary flow of Jeffery nanoliquid past over an extending cylinder under the upshot of heat generation and absorption.

From the above-mentioned literature, it is noted that no attention is paid on the study of MHD flow of *Ag* − *MgO*/water hybrid nanoliquid and heat transition toward the stretching and shrinking surface in the existing of radiation and Joule heating effects. The heat source/sink and slip conditions are also included in the present investigation. To an acquired solution of coupled higher-order non-linear ordinary equations, the homotopy analysis method is hired. The flow and heat transmission of a hybrid nanofluid across an exponentially stretched sheet with mixed convection and Joule heating effects are substantial and advantageous in various engineering, practical and industrial applications. Microelectronics, manufacturing, naval structures, nuclear system cooling, biomedical and drug reduction are only a few of the real-world (practical) and industrial applications of hybrid nanofluid challenges. The skin friction and Nusselt number have been estimated for distinct parameters. Moreover, the behavior of hybrid nanofluid velocity and temperature are calculated in graphical form for various parameters.

Some important applications related to the present physical model are discussed in detail. In the area of PVC (Poly Vinyl Chloride), pipe production, wiredrawing, casting of metals, hot rolling, etc. The concepts of the stretching sheet involve the cooling of continuous strips or filaments by drawing them through a quiescent fluid. In industrial applications, the problem of the heat transfer flow related to the stretching surface has great importance, for instance, in the metallurgical processes, like tinning and annealing of the copper wires, drawing of continuous filaments and manufacturing of the rubber and plastic sheets through the quiescent fluids, fiber spinning and continuous cooling, glass blowing and crystal growing, in addition to extensive applications in various engineering developments like as manufacturing of the paper and foods, stretching of the wire drawing, plastic films, and polymer extrusion, fiber production, and the continuous casting, and many other processes.

## 2. Problem formulation

**Fig 1:**
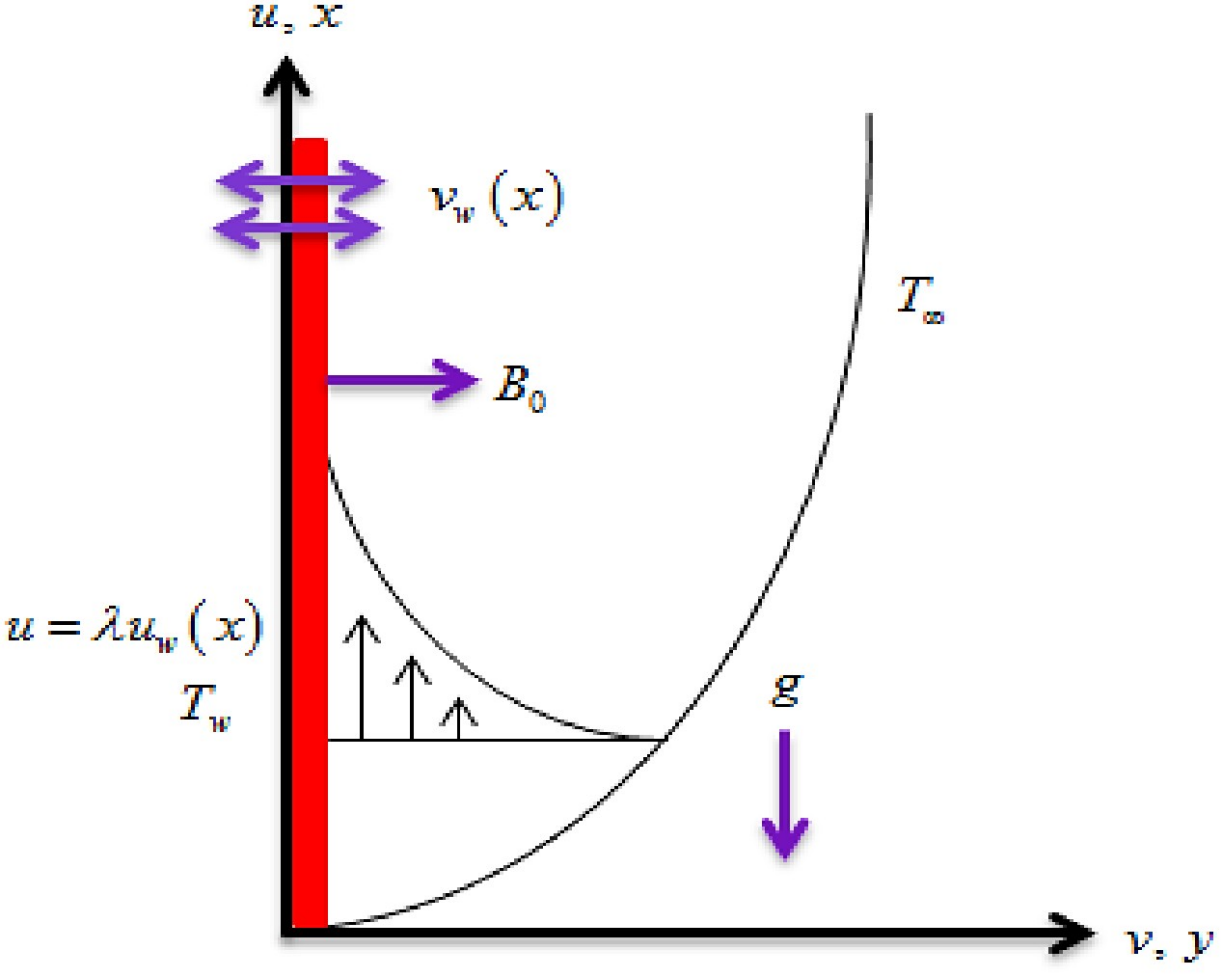
Flow geometry.

The steady MHD flow of hybrid nanofluid flow of *Ag*(silver) and MgO(Magnesium oxide) nanoparticles and water as a base fluid is considered via exponentially stretching sheet. Thermal radiation and Joule heating effects are taken into account. The influence of heat source/sink is used to discuss the generation and absorption of heat phenomena. The slip conditions are taken in the description of the existing model. For the computation of heat transport behavior, the viscous dissipation impact is employed. Along *y*–axis, a magnetic field $B\left(x\right)=B_{0}e^{\frac{x}{L}}$ is employed where *B*_0_ is the applied magnetic field strength. The velocity of the sheet is $u_{w}\left(x\right)=ae^{\frac{x}{L}}$ in which *a* is positive constant. Fig 1 displays the hybrid nanofluid flow mechanism. The temperature at the surface is *T*_*w*_ and ambient temperature is *T*_∞_. In view of the above assumption, the leading equations are [47–51].


$$\frac{\partial u}{\partial x}+\frac{\partial v}{\partial y}=0$$ (1)

$$u\frac{\partial u}{\partial x}+v\frac{\partial u}{\partial y}=\frac{\mu_{hnf}}{\rho_{hnf}}\frac{\partial^{2}u}{\partial y^{2}}-\frac{\sigma_{hnf}B_{0}^{2}}{\rho_{hnf}}u+\frac{{\left(\rho \beta_{T}\right)}_{hnf}}{\rho_{hnf}}g\left(T-T_{\infty }\right),$$ (2)

$$u\frac{\partial T}{\partial x}+v\frac{\partial T}{\partial y}=\left(\frac{k_{hnf}}{{\left(\rho C_{p}\right)}_{hnf}}-\frac{16\sigma^{*}T_{\infty }^{3}}{3k^{*}{\left(\rho C_{p}\right)}_{hnf}}\right)\frac{\partial^{2}T}{\partial y^{2}}+\frac{Q_{0}}{{\left(\rho C_{p}\right)}_{hnf}}\left(T-T_{\infty }\right)+\frac{\sigma_{hnf}}{{\left(\rho C_{p}\right)}_{hnf}}B_{0}^{2}u^{2}+\frac{\mu_{hnf}}{{\left(\rho C_{p}\right)}_{hnf}}{\left(\frac{\partial u}{\partial y}\right)}^{2},$$ (3)

The relevant boundary conditions are

$$\left\{\begin{array}{l} u=\lambda u_{w}\left(x\right)+A_{1}\frac{\partial u}{\partial y},\;\;\;v=v_{w},\;\;T=T_{w}=T_{w}\left(x\right)+B_{1}\frac{\partial T}{\partial y}\;\;\;at\;\;\;y=0,\\ u\to 0,\;\;T\to T_{\infty }\;\;\;as\;\;\;y\to \infty . \end{array}\right\}$$ (4)

The electrical conductivity and heat capacity of the hybrid nanofluid are expressed by *σ*_*hnf*_ and (*ρC*_*p*_)_*hnf*_, respectively, the acceleration due to gravity is *g*, *T* is the temperature, *Q*_0_ is the heat sink/source, *λ* is the stretching parameter, *A*_1_ and *B*_1_ are the slip constants.

The thermophysical properties are defined as

$$\left\{\begin{array}{l} \frac{k_{hnf}}{k_{f}}=\frac{\left(\frac{\phi_{1}ks_{1}+\phi_{2}ks_{2}}{\phi_{hnf}}\right)+2k_{f}+2\left(\phi_{1}ks_{1}+\phi_{2}ks_{2}\right)-2\phi_{hnf}k_{f}}{\left(\frac{\phi_{1}ks_{1}+\phi_{2}\rho s_{2}}{\phi_{hnf}}\right)+2k_{f}-\left(\phi_{1}ks_{1}+\phi_{2}ks_{2}\right)+\phi_{hnf}k_{f}},\\ \rho_{hnf}=\left(1-\phi_{hnf}\right)\rho_{f}+\phi_{2}\rho s_{2}+\phi_{1}\rho s_{1},\;\;\;\phi_{hnf}=\left(\phi_{1}+\phi_{2}\right),\;\;\;\mu_{hnf}=\frac{\mu_{f}}{{\left(1-\phi_{hnf}\right)}^{2.5}},\\ {\left(\rho \beta_{T}\right)}_{hnf}=\left(1-\phi_{hnf}\right){\left(\rho \beta_{T}\right)}_{f}+\left(\rho \beta_{T}\right)\phi_{1}s_{1}+\left(\rho \beta_{T}\right)\phi_{2}s_{2},\\ {\left(\rho C_{p}\right)}_{hnf}=\left(1-\phi_{hnf}\right){\left(\rho C_{p}\right)}_{f}+{\left(\rho C_{p}\right)}_{s1}\phi_{1}+{\left(\rho C_{p}\right)}_{s2}\phi_{2},\\ \;\;\;\frac{\sigma_{hnf}}{\sigma_{f}}=\frac{\left(\frac{\phi_{1}\rho s_{1}+\phi_{2}\rho s_{2}}{\phi_{hnf}}\right)+2\sigma_{f}+2\left(\phi_{1}\rho s_{1}+\phi_{2}\rho s_{2}\right)-2\phi_{hnf}\sigma_{f}}{\left(\frac{\phi_{1}\rho s_{1}+\phi_{2}\rho s_{2}}{\phi_{hnf}}\right)+2\sigma_{f}-\left(\phi_{1}\rho s_{1}+\phi_{2}\rho s_{2}\right)+\phi_{hnf}\sigma_{f}}, \end{array}\right\}$$ (5)

The calculated values of the thermophysical features of the base fluid and nanoparticles are presented in Table 1.

**Table 1:** Thermophysical properties of the base fluid and nanoparticulate.

Property	Pure water	*Ag*(silver)	*MgO*(Magnesium oxide)
*C*_*p*_ (*J*/*kgK*)	4179	235	955
*ρ* (*kg*/*m*^3^)	997.1	10,500	3560
*k* (*W*/*mK*)	0.623	429	45
*β*_*T*_×10^5^ (1/*K*)	21	1.89	1.13

The similarity transformations are defined as [48, 49, 51]:

$$\left\{\begin{array}{l} u=\frac{\partial \psi }{\partial y}=ae^{\frac{x}{L}}f^{\prime }\left(\eta \right),\;\;\;v=-\frac{\partial \psi }{\partial x}=-\sqrt{\frac{av}{2L}}e^{\frac{x}{2L}}\left(f\left(\eta \right)+\eta f^{\prime }\left(\eta \right)\right),\\ \theta \left(\eta \right)=\frac{T-T_{\infty }}{T_{w}-T_{\infty }},\;\;\;\eta =y\sqrt{\frac{a}{2\upsilon L}}e^{\frac{x}{2L}},\;\;\;\psi =\sqrt{2vaL}f\left(\eta \right)e^{\frac{x}{2L}}. \end{array}\right\}$$ (6)

After applying similarity transformations, Eqs (2) and (3) are reduced as:

$$\frac{\mu_{hnf}/\mu_{f}}{\rho_{hnf}/\rho_{f}}f^{\prime \prime \prime }+ff^{\prime \prime }-\frac{\sigma_{hnf}/\sigma_{f}}{\rho_{hnf}/\rho_{f}}Mf^{\prime }-2f^{\prime }+\frac{{\left(\rho \beta_{T}\right)}_{hnf}/{\left(\rho \beta_{T}\right)}_{f}}{\rho_{hnf}/\rho_{f}}2\lambda_{1}\theta =0,$$ (7)

$$\begin{array}{l} \frac{1}{\text{Pr}}\left(\frac{k_{hnf}/k_{f}}{{\left(\rho C_{p}\right)}_{hnf}/{\left(\rho C_{p}\right)}_{f}}+\frac{4}{3}\frac{{\left(\rho C_{p}\right)}_{f}}{{\left(\rho C_{p}\right)}_{hnf}}Rd\right)\theta^{\prime \prime }+\frac{1}{{\left(\rho C_{p}\right)}_{hnf}/{\left(\rho C_{p}\right)}_{f}}Q\theta \\ +f\theta^{\prime }+\frac{\mu_{hnf}/\mu_{f}}{{\left(\rho C_{p}\right)}_{hnf}/{\left(\rho C_{p}\right)}_{f}}Ec{f^{\prime \prime }}^{2}+\frac{\sigma_{hnf}/\sigma_{f}}{{\left(\rho C_{p}\right)}_{hnf}/{\left(\rho C_{p}\right)}_{f}}MEc{f^{\prime }}^{2}=0, \end{array}$$ (8)

The transformed conditions are

$$\left\{\begin{array}{l} f\left(\eta \right)=S,\;\;\;f^{\prime }\left(\eta \right)=\lambda +Af^{\prime \prime }\left(\eta \right),\;\;\;\theta \left(\eta \right)=1+B\theta^{\prime }\left(\eta \right)\;\;\;at\;\;\;\eta =0,\\ f^{\prime }\left(\eta \right)\to 0,\;\;\;\theta \left(\eta \right)\to 0\;\;\;as\;\;\;\eta \to \infty . \end{array}\right\}$$ (9)

In the above equations, *η* is the similarity variable, the radiation parameter is denoted by $Rd=\frac{4\sigma^{*}T_{\infty }^{3}}{k^{*}k_{f}}$, $Q=\frac{2LQ_{0}}{u_{w}{\left(\rho C_{p}\right)}_{f}}$ is the dimensionless heat generation term, the Prandtl number is symbolized by $\text{Pr}=\frac{{\left(\mu C_{p}\right)}_{f}}{k_{f}}$, $Ec=\frac{u_{w}^{2}}{{\left(C_{p}\right)}_{f}\left(T_{w}-T_{\infty }\right)}$ is used for Eckert number, the magnetic field parameter is $M=\frac{2L\sigma_{f}B_{0}^{2}}{ae^{\frac{x}{L}}\rho_{f}}$, and $\lambda_{1}=\frac{L{\left(\beta_{T}\right)}_{f}g\left(T_{w}-T_{\infty }\right)}{u_{w}^{2}}$ is for mixed convection parameter. The dimensionless velocity and temperature are signified by *f* and *θ*. The dimensionless velocity slip parameter is $A=A_{1}e^{\frac{x}{2L}}\sqrt{\frac{a}{2\upsilon_{f}L}}$, the dimensionless thermal slip parameter is $B=B_{1}e^{\frac{x}{2L}}\sqrt{\frac{a}{2\upsilon_{f}L}}$, and the suction/injection parameter is denoted by $S=-\frac{-v_{0}}{\sqrt{\frac{\upsilon_{f}a}{2L}}}$. Two cases arises on *S*, for mass suction *S* > 0, (*ν*_0_ < 0), and for mass injection *S* < 0, (*ν*_0_ > 0).

Mathematically, the significant physical quantities are stated as:

$$C_{f}=\frac{\tau_{w}}{\rho_{f}u_{w}^{2}},\;\;\;Nu_{x}=\frac{Lq_{w}}{k_{f}\left(T_{w}-T_{\infty }\right)},$$ (10)

where

$$\tau_{w}=\mu_{hnf}{\left(\frac{\partial u}{\partial y}\right)}_{y=0},\;\;\;q_{w}=k_{hnf}{\left(\frac{\partial T}{\partial y}\right)}_{y=0}.$$ (11)

By using the similarity transformations defined in (6), the dimensionless form are:

$$C_{fx}{\text{Re}}_{x}^{1/2}=\frac{1}{{\left(1-\phi_{1}\right)}^{2.5}{\left(1-\phi_{2}\right)}^{2.5}}f^{\prime \prime }\left(0\right),\;\;\;Nu_{x}{\text{Re}}_{x}^{-1/2}=-\left(\frac{k_{hnf}}{k_{f}}+\frac{4}{3}Rd\right)\theta^{\prime }\left(0\right).$$ (12)

## 3. Solution of the problem

The analytical solution of the higher-order ordinary differential equations is gained through the homotopy analysis method via Mathematica 10. The initial guesses and linear operators are defined as:

$$f_{0}(\eta )=S+\frac{\lambda }{1+A}\left(1-e^{-\eta }\right),\;\;\;\theta_{0}(\eta )=\frac{\lambda }{1+B}e^{-\eta },$$ (13)

$$L_{f}=f^{\prime \prime \prime }-f^{\prime },\;\;\;L_{\theta }={\theta^{\prime }}^{\prime }-\theta .$$ (14)

with

$$L_{f}[C_{1}+C_{2}\;\text{exp}(\eta )+C_{3}\;\text{exp}(-\eta )]=0,\;\;\;L_{\theta }[C_{4}\;\text{exp}(\eta )+C_{5}\;\text{exp}(-\eta )]=0,$$ (15)

where *C*_*i*_(*i* = 1 − 5) are the arbitrary constants.

### 3.1 Zeroth order deformation problem

The zero-order deformation for the present hybrid nanofluid model is:

$$(1-q)L_{f}[f(\eta ,q)-f_{0}(\eta )]=qh_{f}N_{f}[f(\eta ,q),\theta (\eta ,q)],$$ (16)

$$(1-q)L_{\theta }[\theta (\eta ,q)-\theta_{0}(\eta )]=qh_{\theta }N_{\theta }[F(\eta ,q),\theta (\eta ,q)],$$ (17)

In the present case *N*_*f*_ and *N*_*θ*_ are the nonlinear operators and given as:

$$N_{f}[f(\eta ,q),\theta (\eta ,q)]=\frac{\mu_{hnf}/\mu_{f}}{\rho_{hnf}/\rho_{f}}\frac{\partial^{3}f(\eta ,q)}{\partial \eta^{3}}-\left(\frac{\sigma_{hnf}/\sigma_{f}}{\rho_{hnf}/\rho_{f}}M+2\right)\frac{\partial f(\eta ,q)}{\partial \eta }+\frac{{\left(\rho \beta_{T}\right)}_{hnf}/{\left(\rho \beta_{T}\right)}_{f}}{\rho_{hnf}/\rho_{f}}2\lambda_{1}\theta (\eta ,q),$$ (18)

$$\begin{array}{l} N_{\theta }[f(\eta ,q),\theta (\eta ,q)]=\frac{1}{\text{Pr}}\left(\frac{k_{hnf}/k_{f}}{{\left(\rho C_{p}\right)}_{hnf}/{\left(\rho C_{p}\right)}_{f}}+\frac{4}{3}\frac{{\left(\rho C_{p}\right)}_{f}}{{\left(\rho C_{p}\right)}_{hnf}}Rd\right)\frac{\partial^{2}\theta (\eta ,q)}{\partial \eta^{2}}+f(\eta ,q)\frac{\partial \theta (\eta ,q)}{\partial \eta }\\ \frac{1}{{\left(\rho C_{p}\right)}_{hnf}/{\left(\rho C_{p}\right)}_{f}}Q\theta (\eta ,q)+\frac{\mu_{hnf}/\mu_{f}}{{\left(\rho C_{p}\right)}_{hnf}/{\left(\rho C_{p}\right)}_{f}}Ec\frac{\partial^{2}f(\eta ,q)}{\partial \eta^{2}}+\frac{\sigma_{hnf}/\sigma_{f}}{{\left(\rho C_{p}\right)}_{hnf}/{\left(\rho C_{p}\right)}_{f}}MEc{\left(\frac{\partial f(\eta ,q)}{\partial \eta }\right)}^{2}, \end{array}$$ (19)

$$f(0,q)=S,\;\;\;f^{\prime }(0,q)=\lambda +Af^{\prime \prime }\left(0,q\right),\;\;\;f^{\prime }(\infty ,q)=0,$$ (20)

$$\theta (0,q)=1+B\theta^{\prime }\left(0,q\right),\;\;\;\theta (\infty ,q)=0.$$ (21)

For *q* = 0 and *q* = 1, then (16) and (17) become as:

$$q=0\Rightarrow f(\eta ,0)=f_{0}(\eta ),\;\;\;and\;\;\;q=1\Rightarrow f(\eta ,1)=f(\eta ).$$ (22)

$$q=0\Rightarrow \theta (\eta ,0)=\theta_{0}(\eta ),\;\;\;and\;\;\;q=1\Rightarrow \theta (\eta ,1)=\theta (\eta ).$$ (23)

Taylor expansion is employed on (22) and (23), it is obtained that:

$$f(\eta ,q)=f_{0}(\eta )+\sum_{m=1}^{\infty }f_{m}(\eta )q^{m},\;\;\;f_{m}(\eta )=\frac{1}{m!}\frac{\partial^{m}f(\eta ,q)}{\partial \eta^{m}}{|}_{q=0}.$$ (24)

$$\theta (\eta ,q)=\theta_{0}(\eta )+\sum_{m=1}^{\infty }\theta_{m}(\eta )q^{m},\;\;\;\theta_{m}(\eta )=\frac{1}{m!}\frac{\partial^{m}\theta (\eta ,q)}{\partial \eta^{m}}{|}_{q=0}.$$ (25)

By taking *q* = 1 in (24) and (25), the convergence of the series is found as:

$$f(\eta )=f_{0}(\eta )+\sum_{m=1}^{\infty }f_{m}(\eta ).$$ (26)

$$\theta (\eta )=\theta_{0}(\eta )+\sum_{m=1}^{\infty }\theta_{m}(\eta ).$$ (27)

### 3.2 m^*th*^-order deformation problem

Now the m^*th*^ deformation of the problem is explained as:

$$L_{f}[f_{m}(\eta )-\eta_{m}f_{m-1}(\eta )]=h_{f}R_{m}^{f}m(\eta ),$$ (28)

$$L_{\theta }[\theta_{m}(\eta )-\eta_{m}\theta_{m-1}(\eta )]=h_{\theta }R_{m}^{\theta }m(\eta ),$$ (29)

$$f_{m}(0)=0,f_{m}(\infty )=0,$$ (30)

$$\theta_{m}(0)=0,\;\;\;\theta_{m}(\infty )=0,$$ (31)

The $R_{m}^{f}m(\eta )$ and $R_{m}^{\theta }m\left(\eta \right)$ are defined below:

$$R_{m}^{f}(\eta )=\frac{\mu_{hnf}/\mu_{f}}{\rho_{hnf}/\rho_{f}}f_{m-1}^{\prime \prime \prime }-\left(\frac{\sigma_{hnf}/\sigma_{f}}{\rho_{hnf}/\rho_{f}}M+2\right)f_{m-1}^{\prime }+\frac{{\left(\rho \beta_{T}\right)}_{hnf}/{\left(\rho \beta_{T}\right)}_{f}}{\rho_{hnf}/\rho_{f}}2\lambda_{1}\theta_{m},$$ (32)

$$\begin{array}{l} R_{m}^{\theta }(\eta )=\frac{1}{\text{Pr}}\left(\frac{k_{hnf}/k_{f}}{{\left(\rho C_{p}\right)}_{hnf}/{\left(\rho C_{p}\right)}_{f}}+\frac{4}{3}\frac{{\left(\rho C_{p}\right)}_{f}}{{\left(\rho C_{p}\right)}_{hnf}}Rd\right)\theta_{m-1}^{\prime \prime }+\frac{1}{{\left(\rho C_{p}\right)}_{hnf}/{\left(\rho C_{p}\right)}_{f}}Q\theta_{m}\\ +\sum_{k=0}^{m-1}f_{m-1-k}\theta_{k}^{\prime }+\frac{\mu_{hnf}/\mu_{f}}{{\left(\rho C_{p}\right)}_{hnf}/{\left(\rho C_{p}\right)}_{f}}Ecf_{m-1}^{\prime \prime }+\frac{\sigma_{hnf}/\sigma_{f}}{{\left(\rho C_{p}\right)}_{hnf}/{\left(\rho C_{p}\right)}_{f}}MEc\sum_{k=0}^{m-1}{\left(f_{m-1}^{\prime }\right)}^{2}, \end{array}$$ (33)

The general solution is achieved through the use of particular solutions:

$$f_{m}(\eta )=f_{m}^{*}(\eta )+C_{2}\;\text{exp}(-\eta )+C_{3}\;\text{exp}(\eta ).$$ (34)

$$\theta_{m}(\eta )=\theta_{m}^{*}(\eta )+C_{6}\;\text{exp}(-\eta )+C_{7}\;\text{exp}(\eta ),$$ (35)

## 4. Convergence of HAM

**Fig 2:**
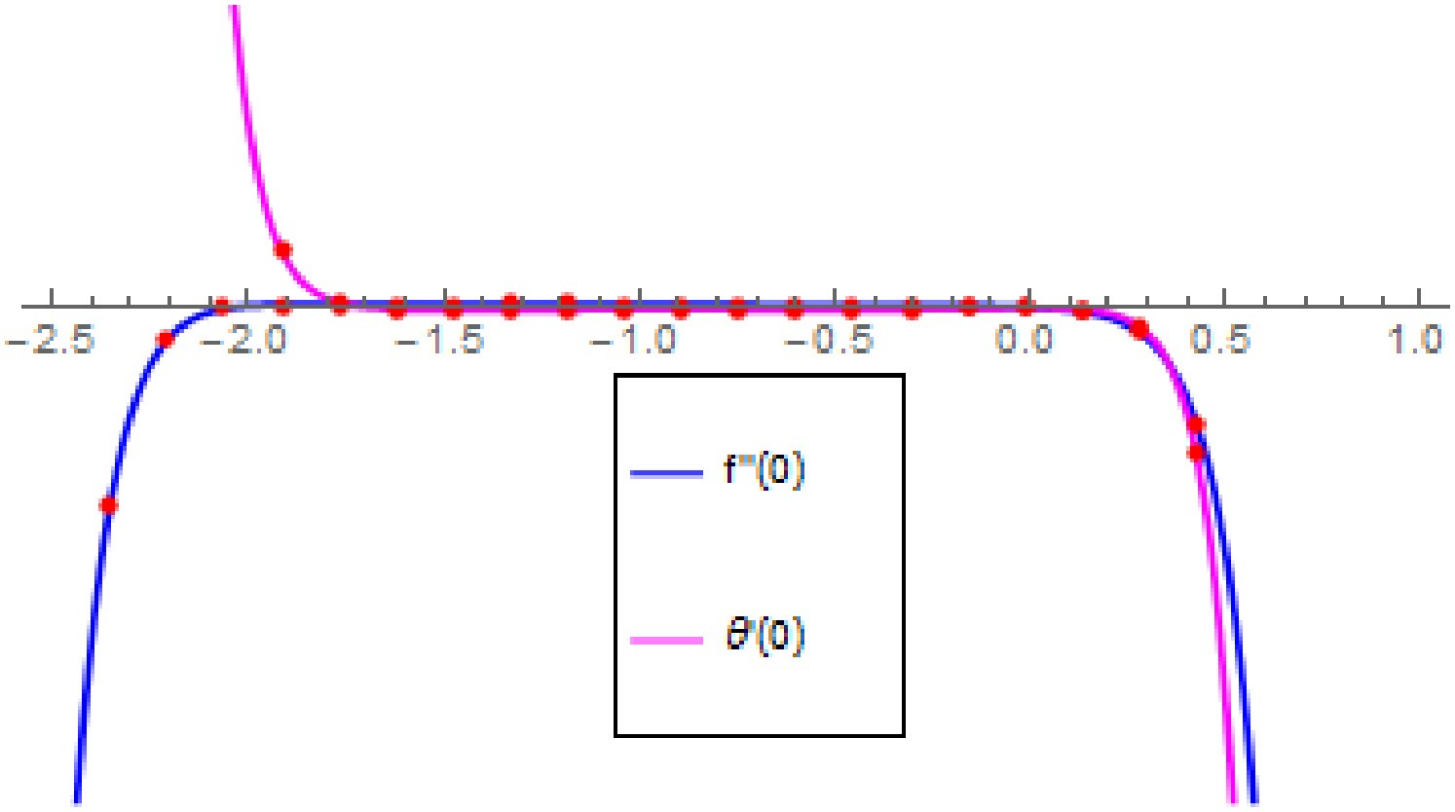
*h* − *curves* for *f*″(0) and *θ*′(0).

Liao [52] was the first mathematician who introduced the *h* − curves for the series solution of the problems in order to attain the accurate and convergent solution of the problem. The solution of the problem in the HAM *h* − curves is also known as the convergence controlling parameters. Form the valid region these optimum values are chosen in a straight line that is parallel to the horizontal axis (see in Fig 2) to control the convergent solution of the problem. The valid region of the *h* − curves are stated for the present case. The different parameters are used for the preparation of *h* − curves. Therefore, the acceptable *h* − curves for *f*″(0) and *θ*′(0) are drawn in the following range -2.5 ≤ h ≥ 1.0 in Fig 2.

## 5. Results and discussion

**Fig 3:**
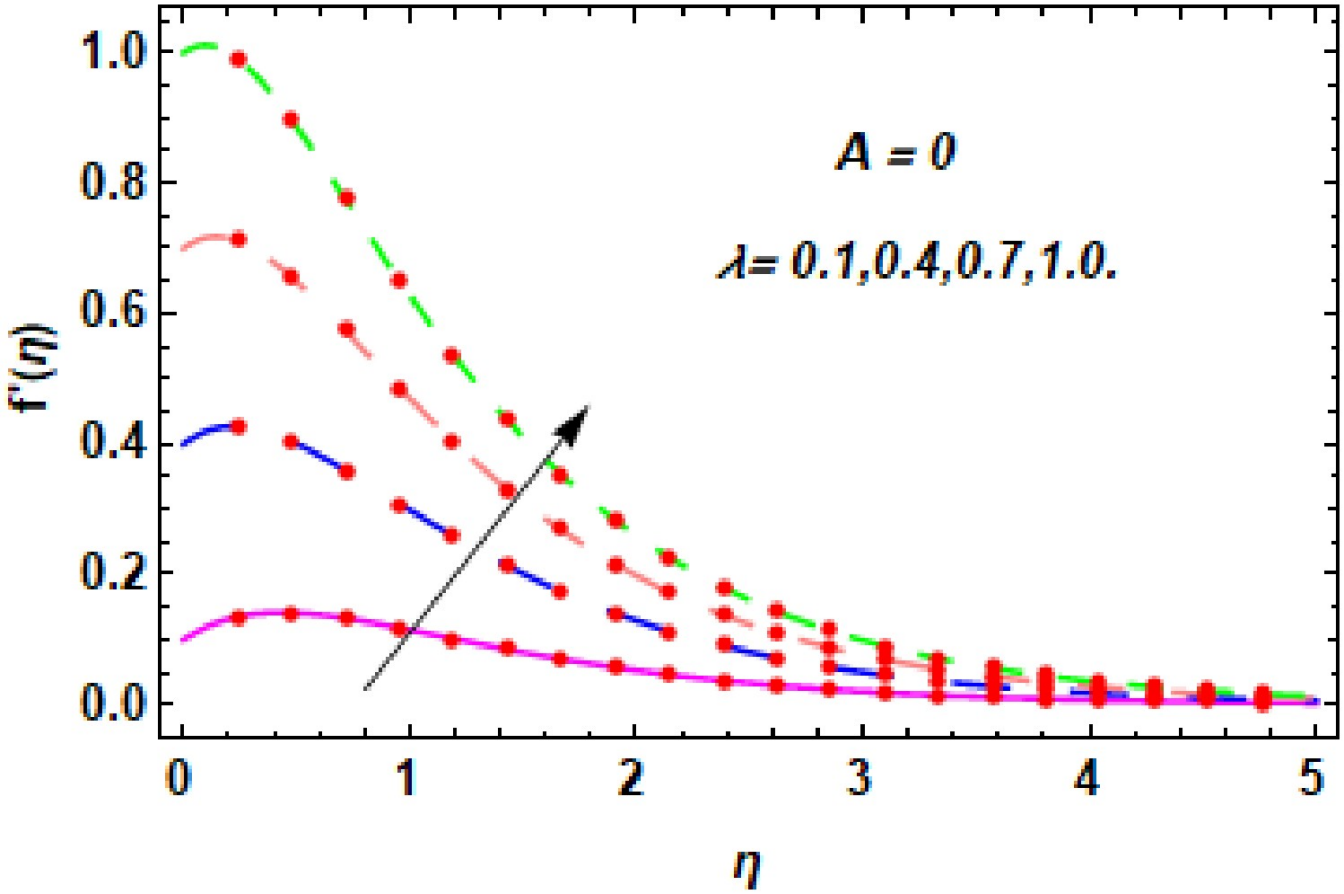
The nature of velocity field for *λ*.

**Fig 4:**
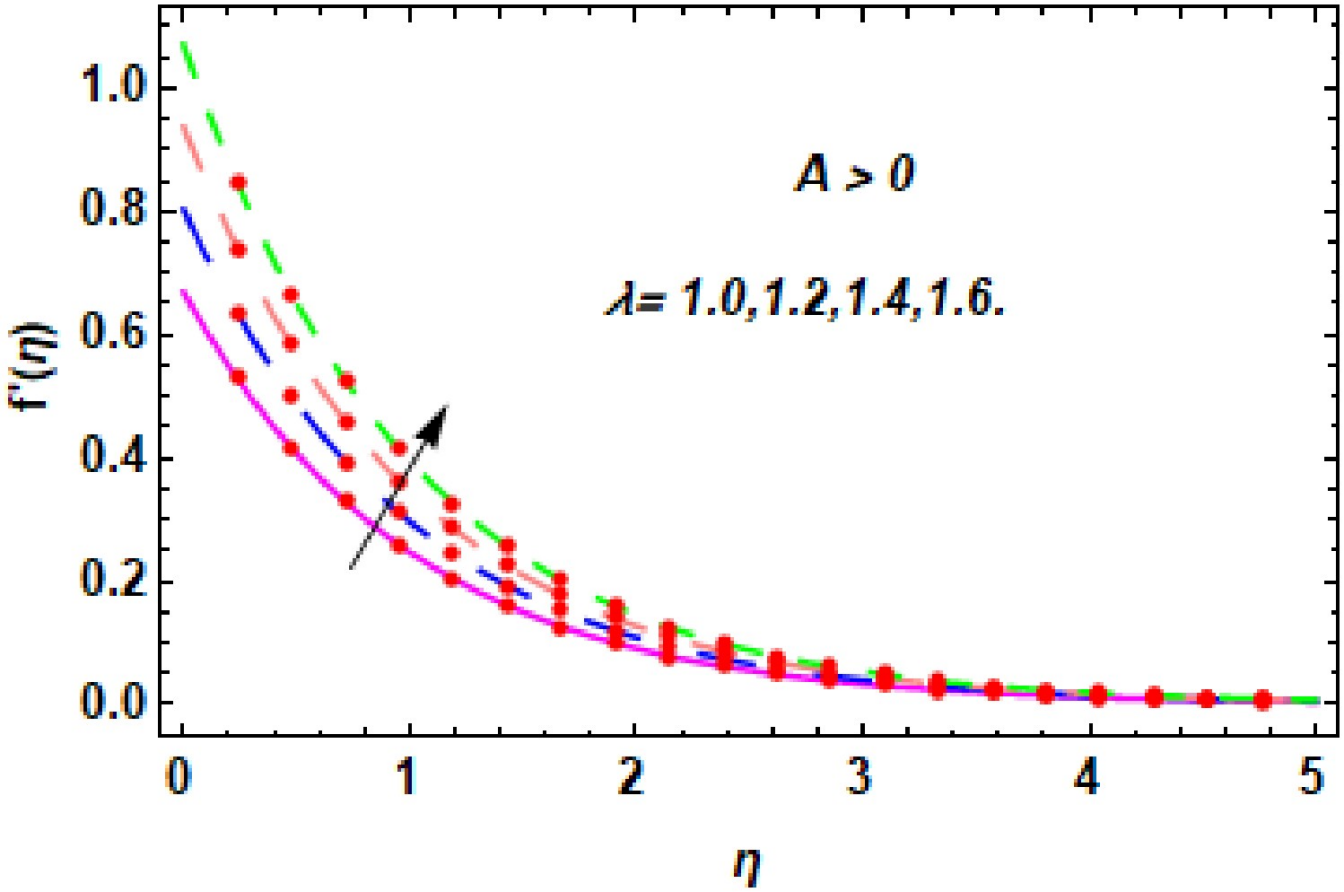
The nature of velocity field for *λ*.

**Fig 5:**
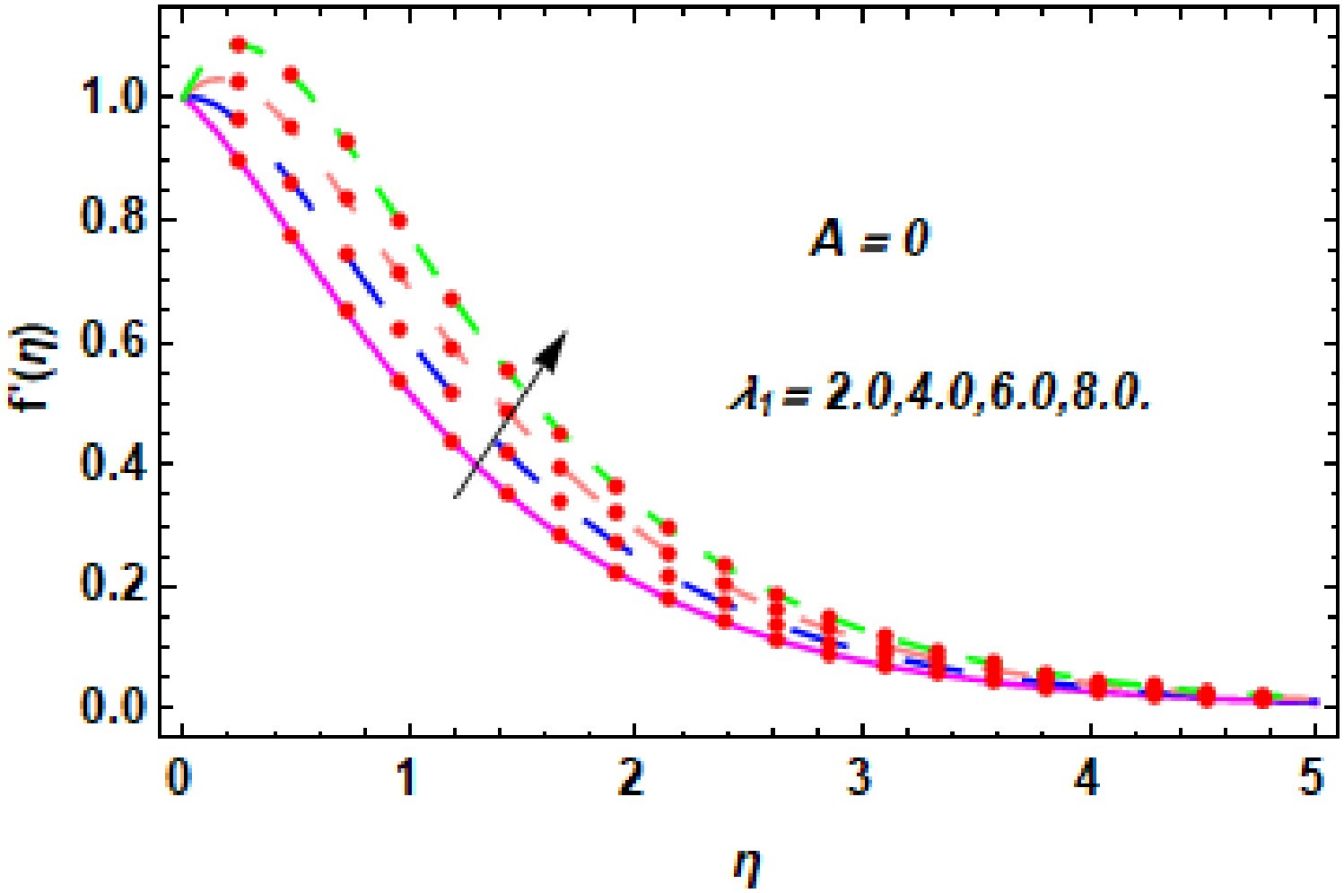
The nature of velocity field for *λ*_1_.

**Fig 6:**
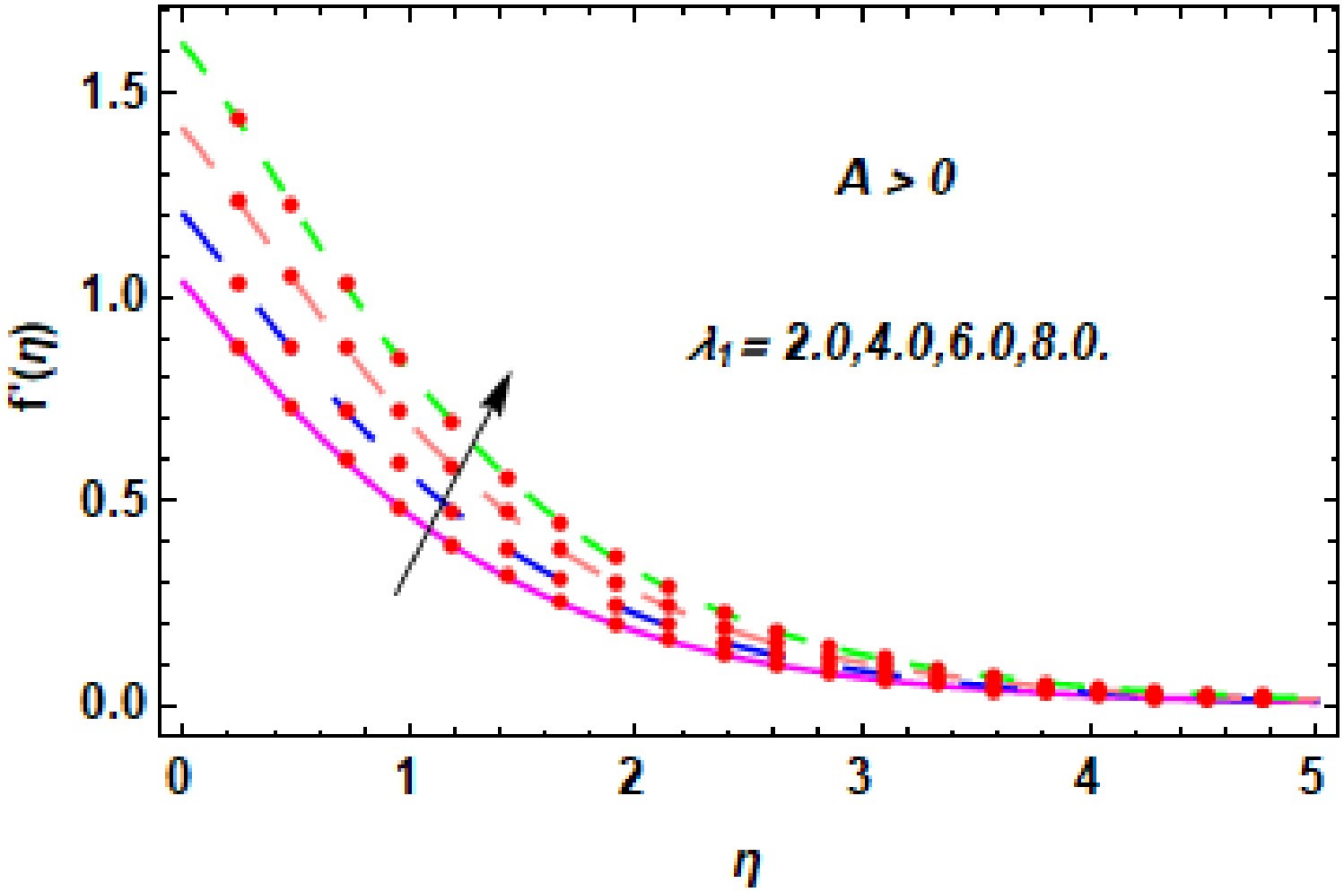
The nature of velocity field for *λ*_1_.

**Fig 7:**
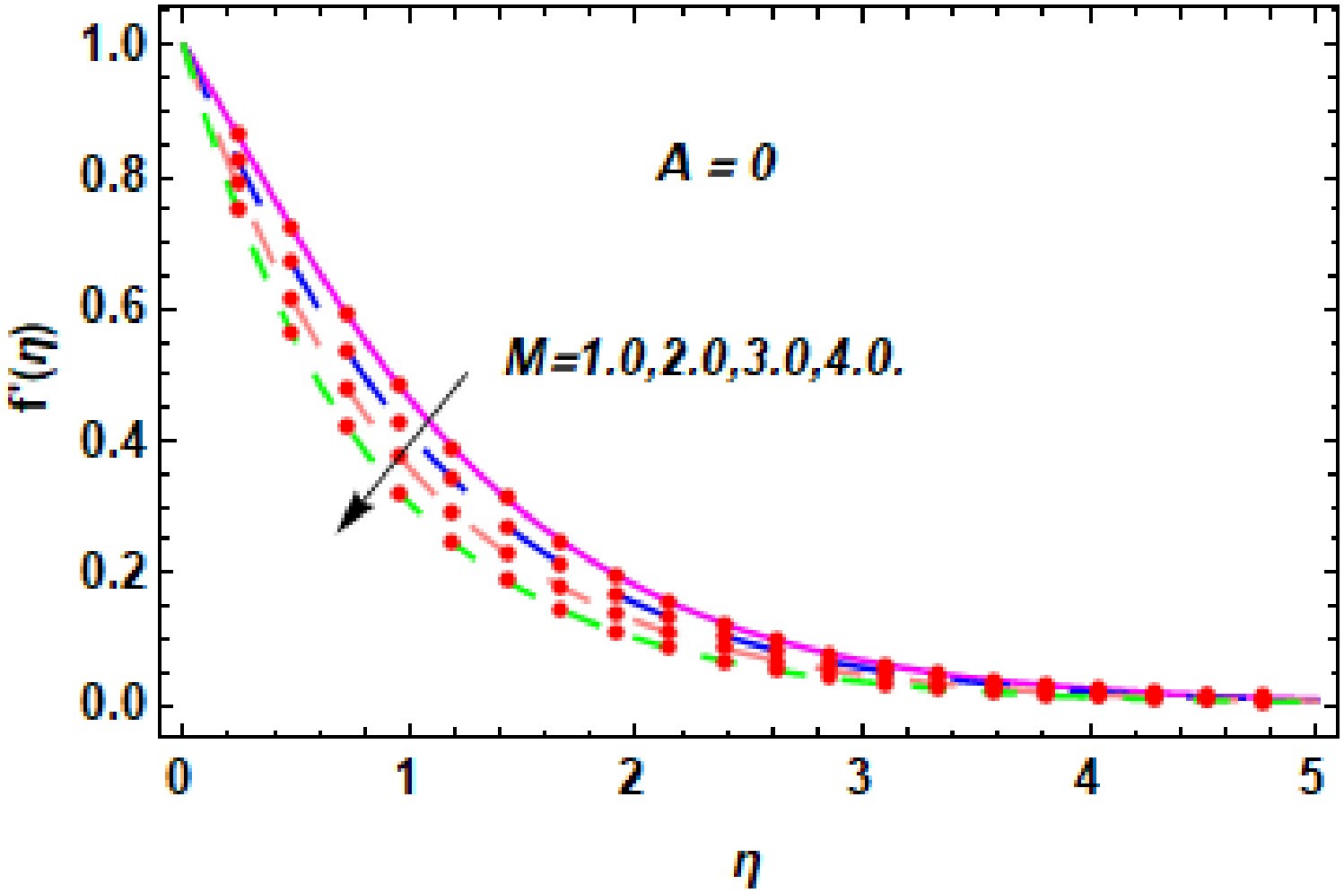
The nature of velocity field for *M*.

**Fig 8:**
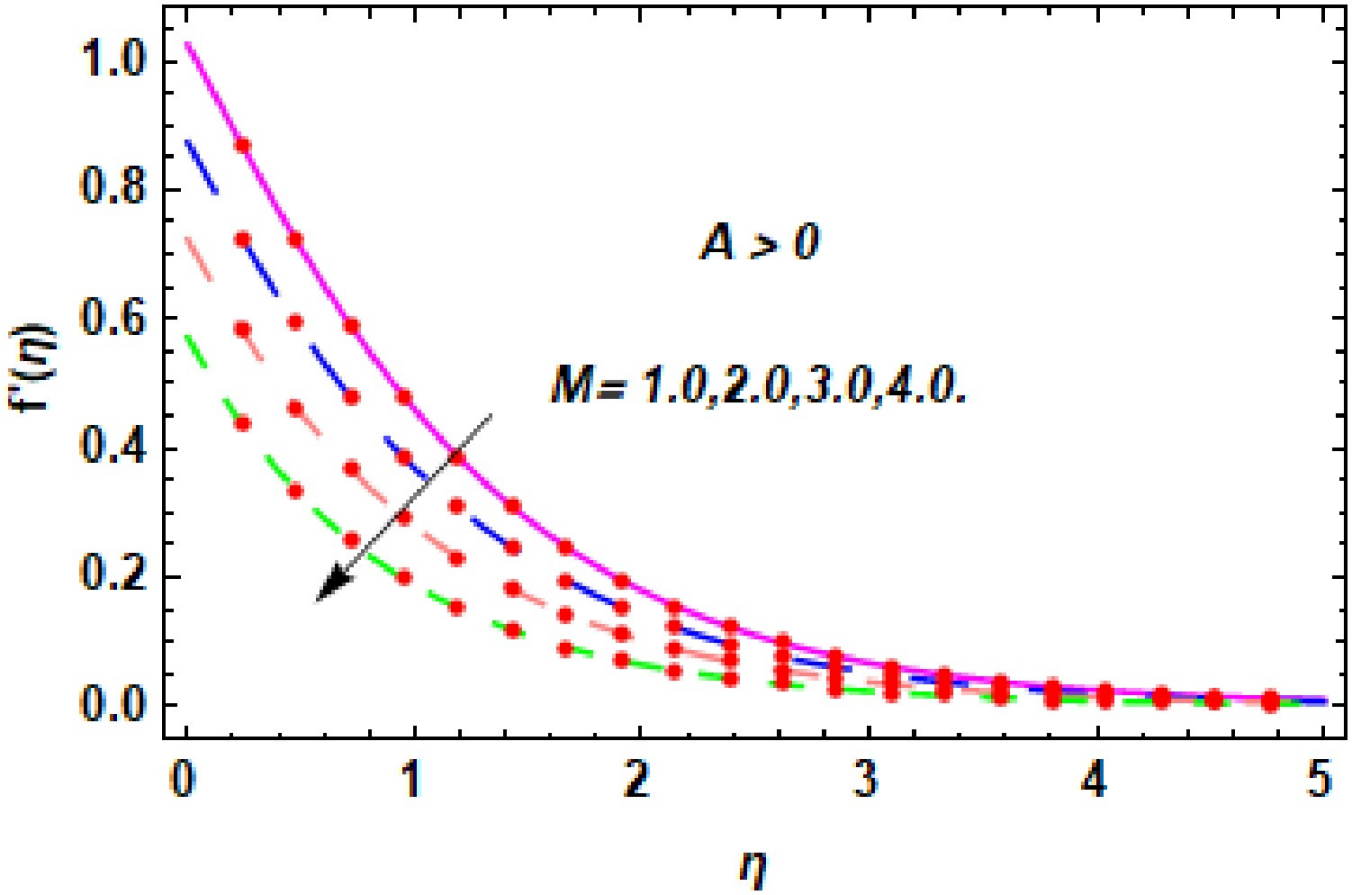
The nature of velocity field for *M*.

**Fig 9:**
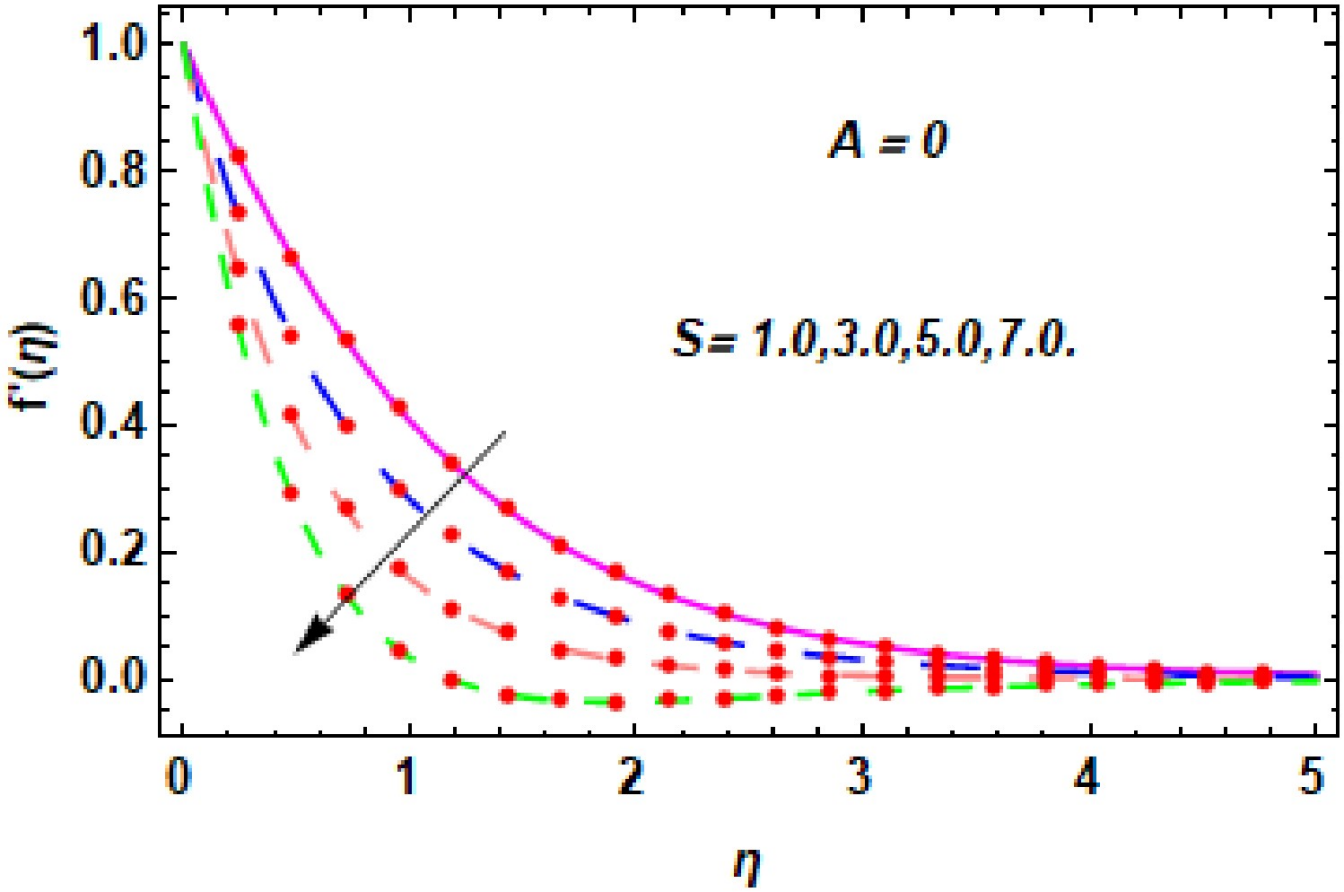
The nature of velocity field for *S*.

**Fig 10:**
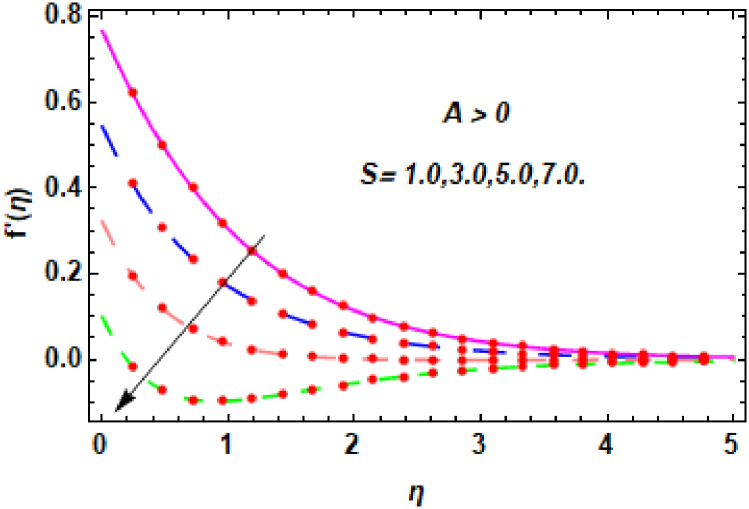
The nature of velocity field for *S*.

**Fig 11:**
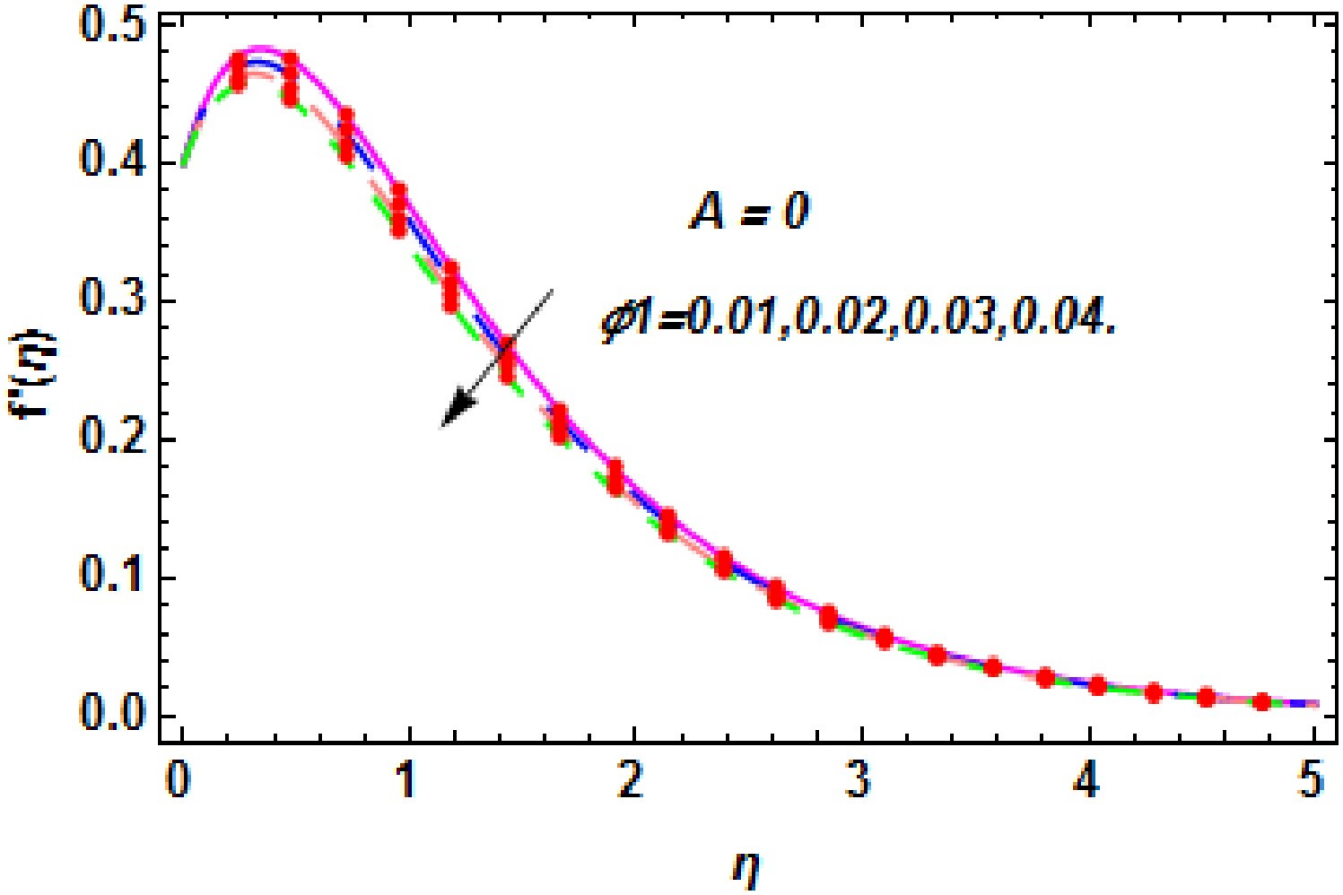
The nature of velocity field for *ϕ*_1_.

**Fig 12:**
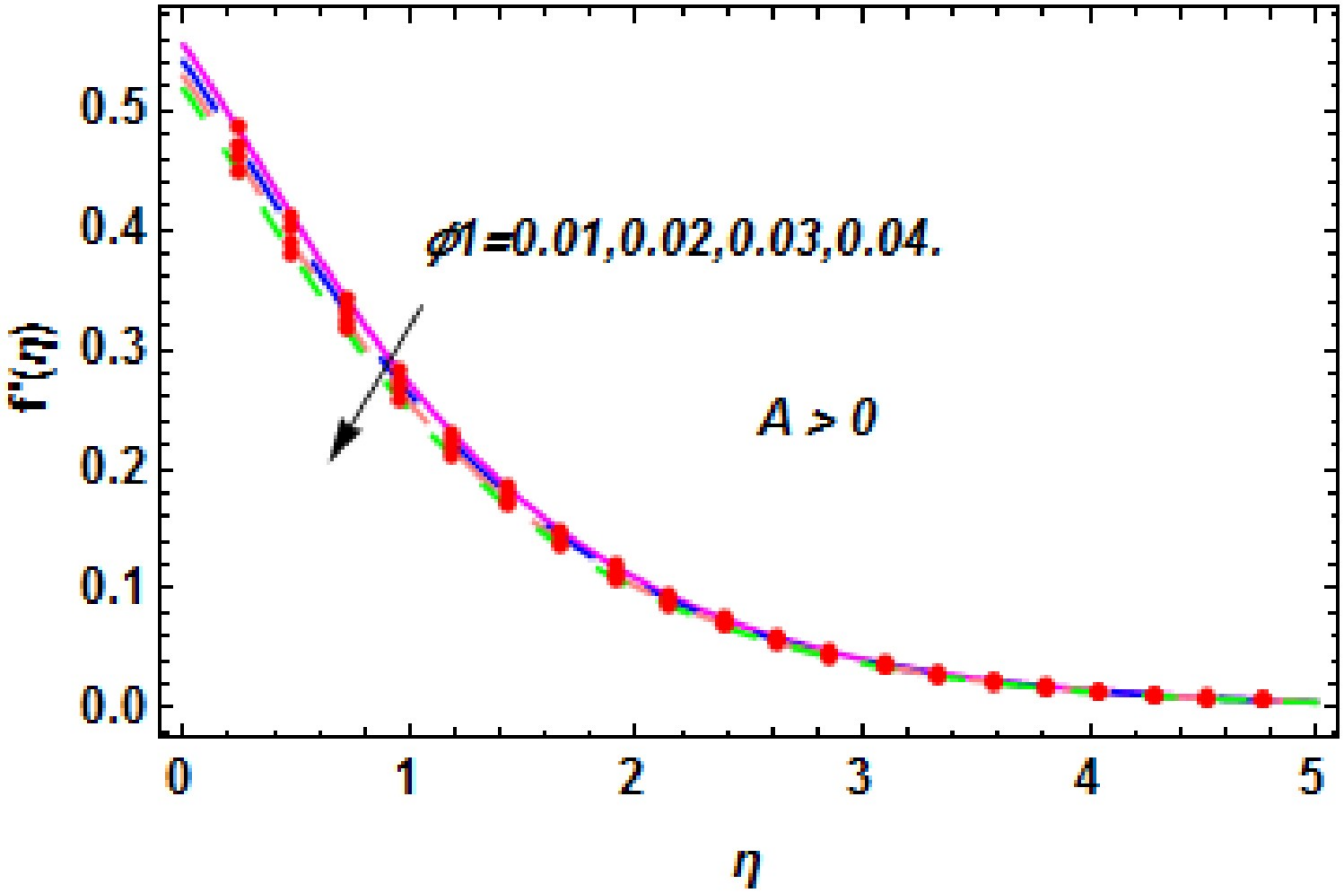
The nature of velocity field for *ϕ*_1_.

**Fig 13:**
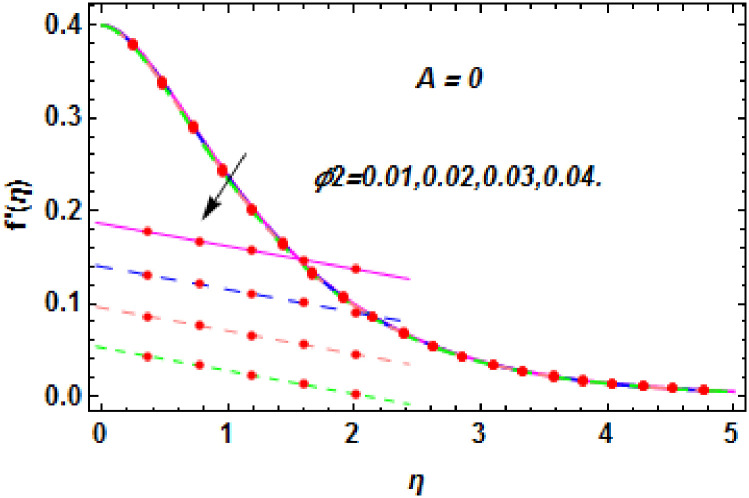
The nature of velocity field for *ϕ*_2_.

**Fig 14:**
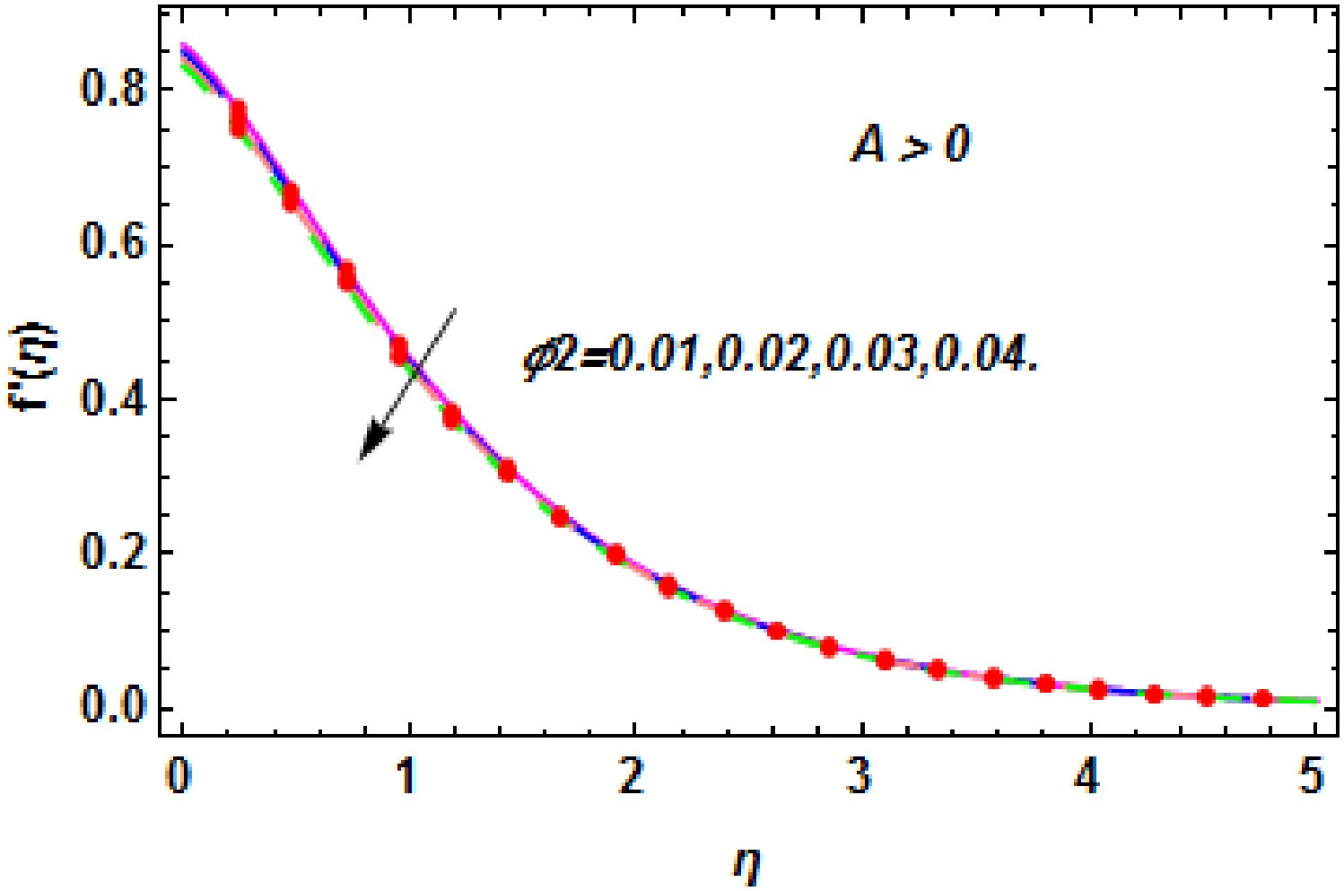
The nature of velocity field for *ϕ*_2_.

**Fig 15:**
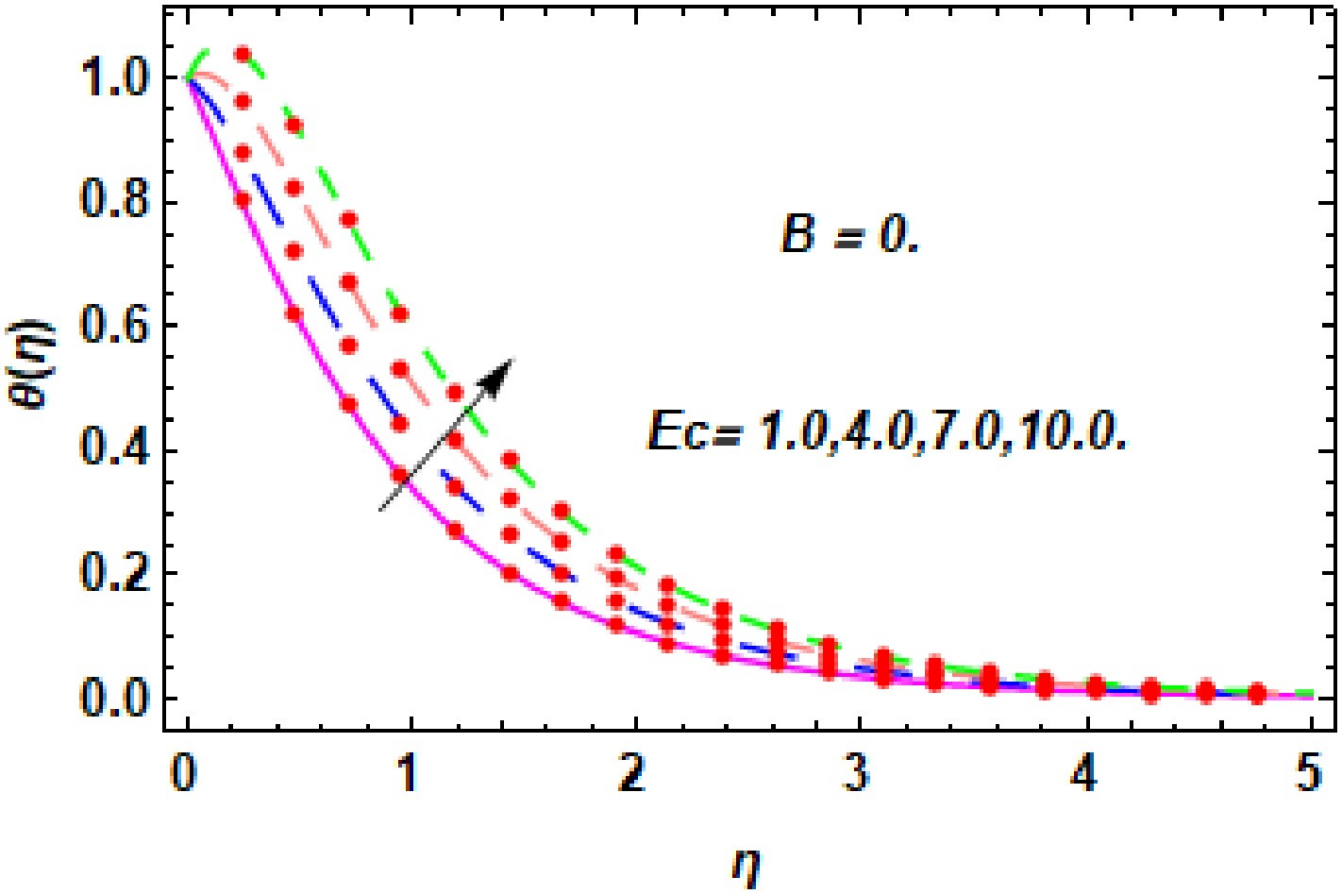
The nature of energy field for *Ec*.

**Fig 16:**
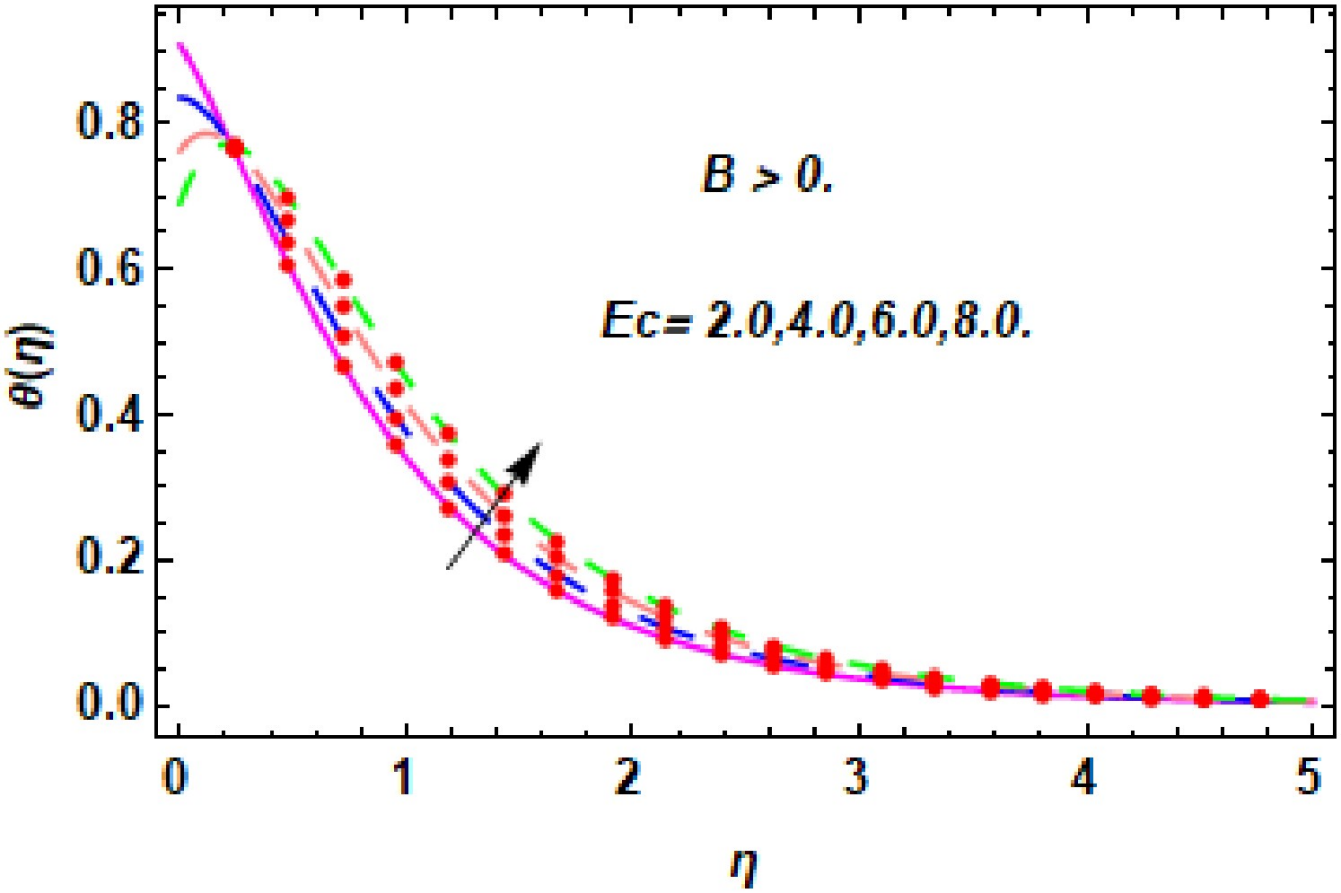
The nature of energy field for *Ec*.

**Fig 17:**
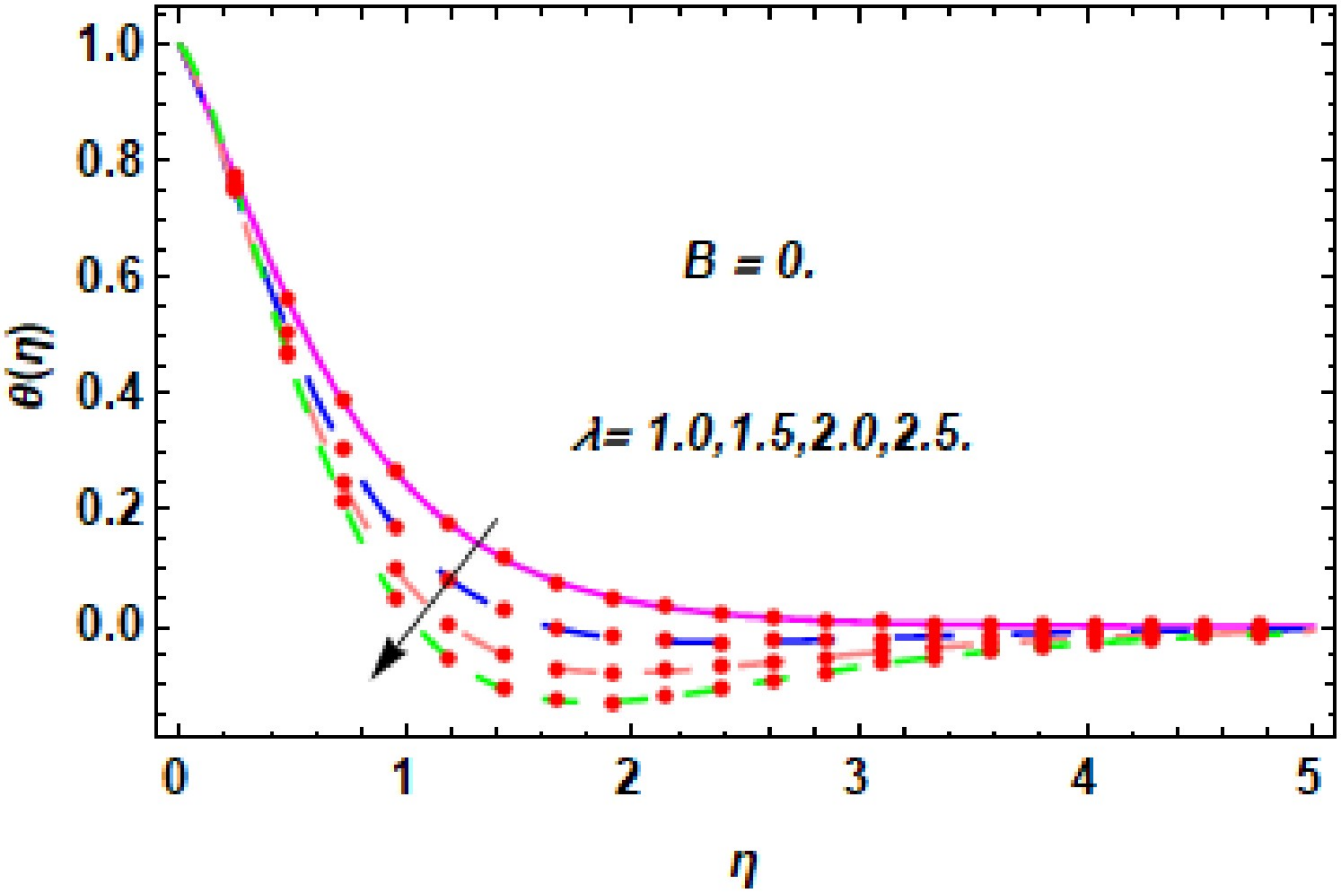
The nature of energy field for *λ*.

**Fig 18:**
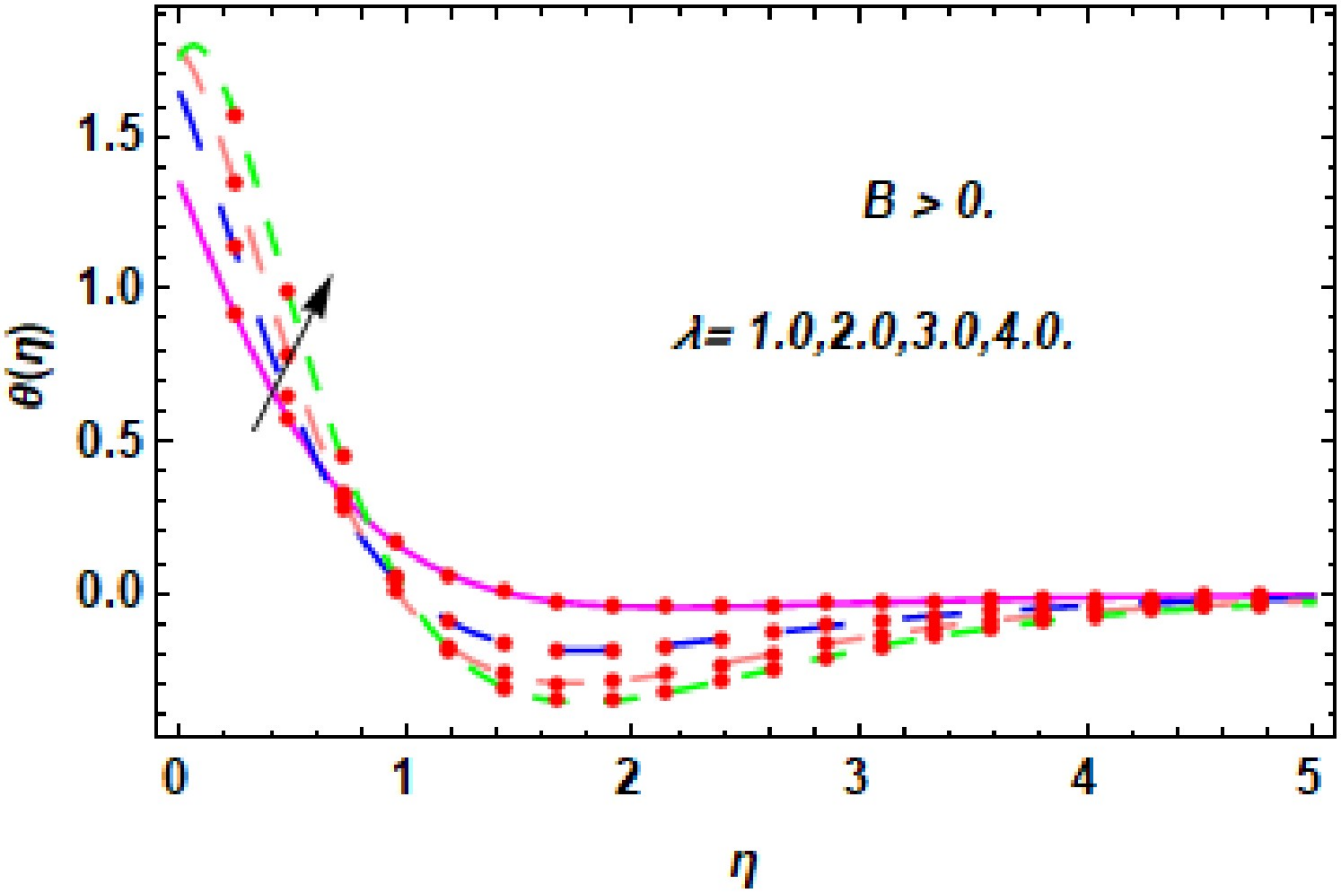
The nature of energy field for *λ*.

**Fig 19:**
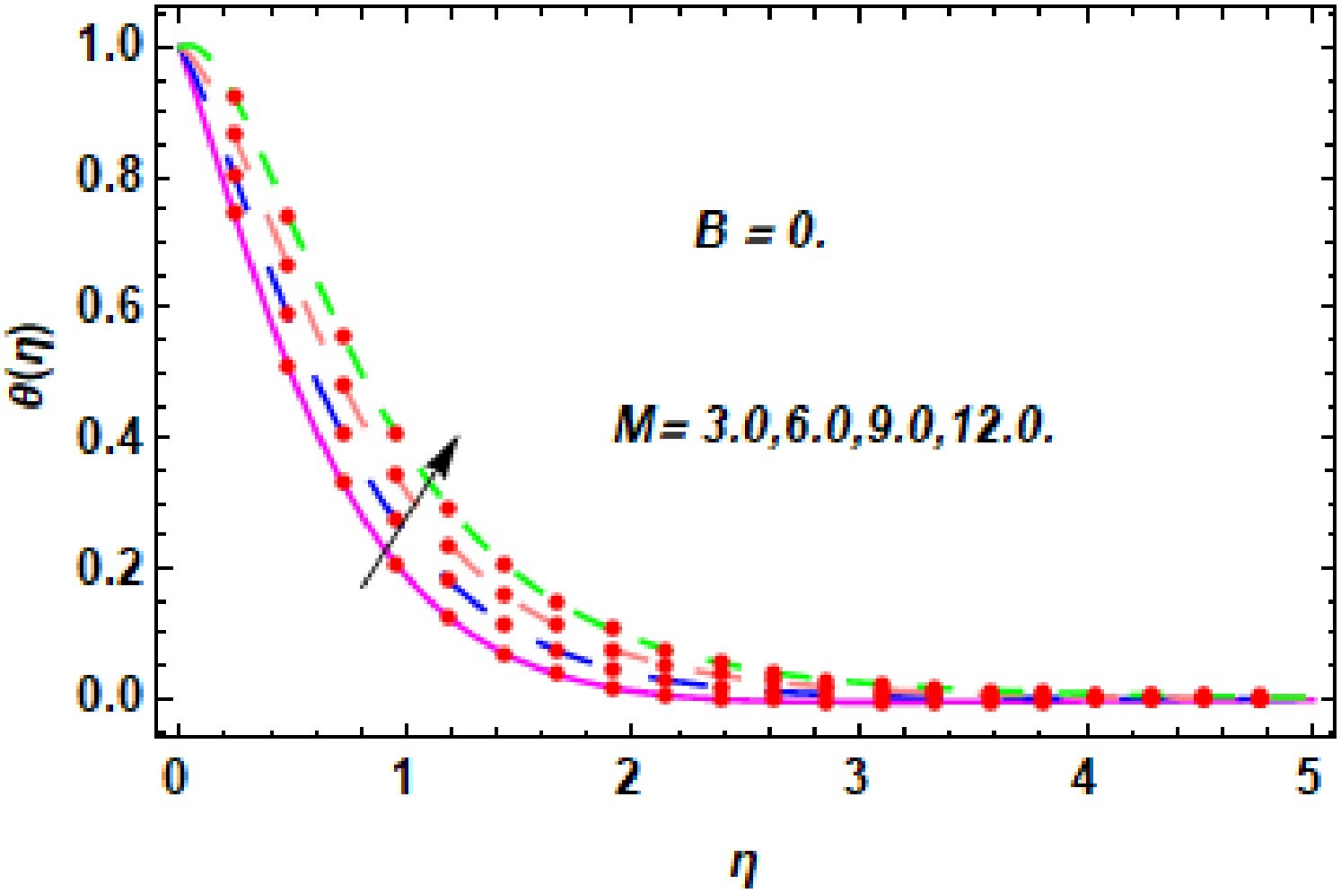
The nature of energy field for *M*.

**Fig 20:**
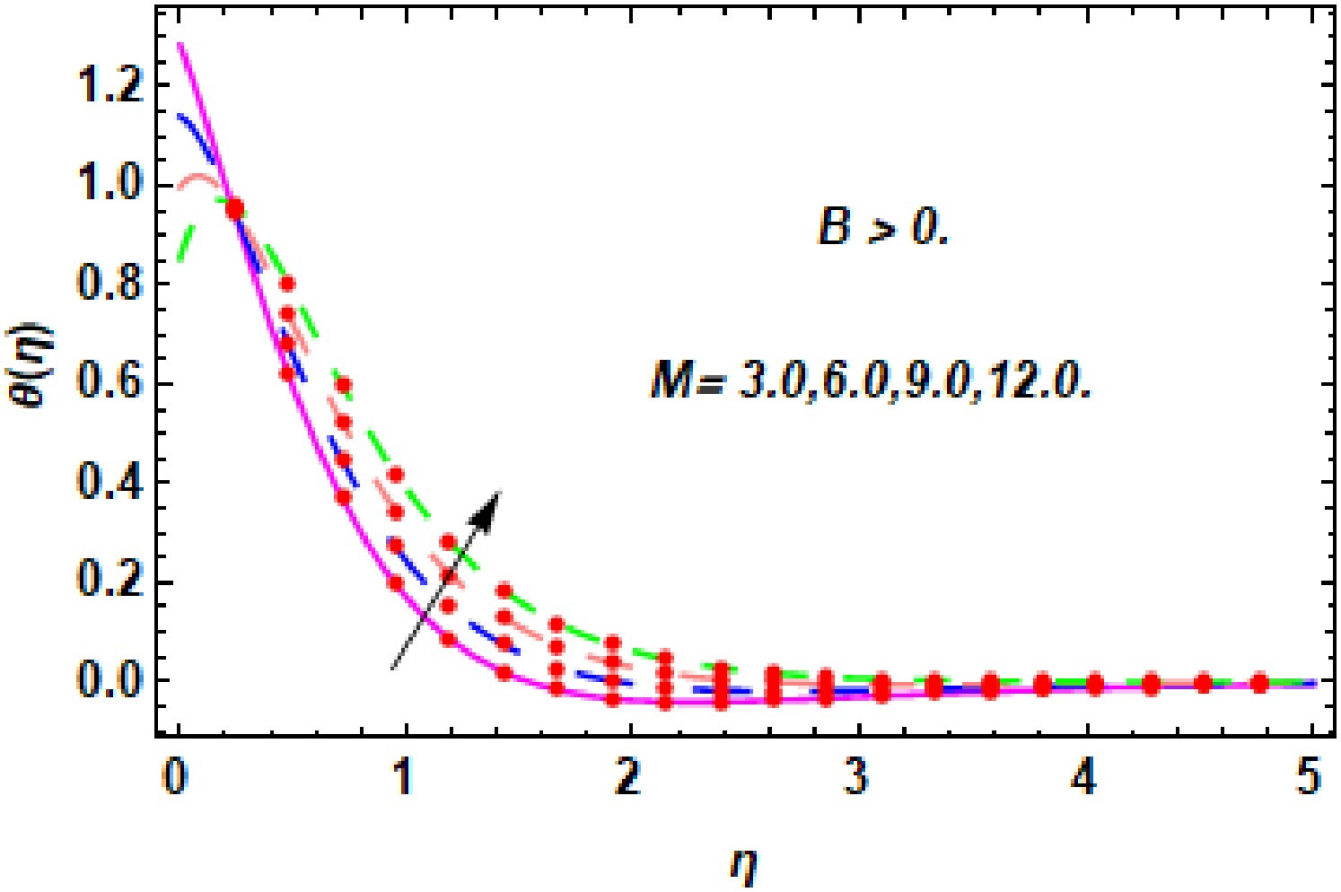
The nature of energy field for *M*.

**Fig 21:**
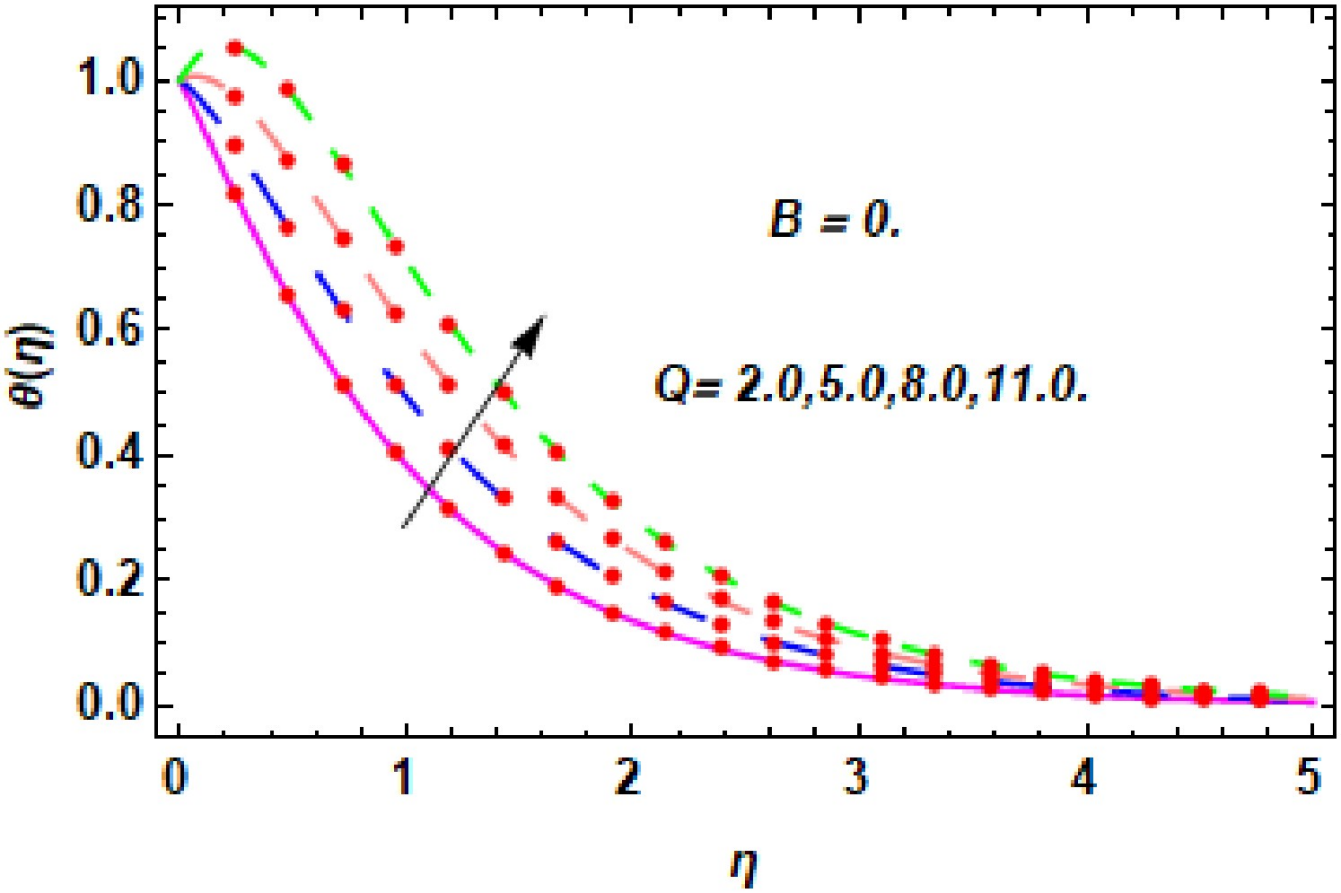
The nature of energy field for *Q*.

**Fig 22:**
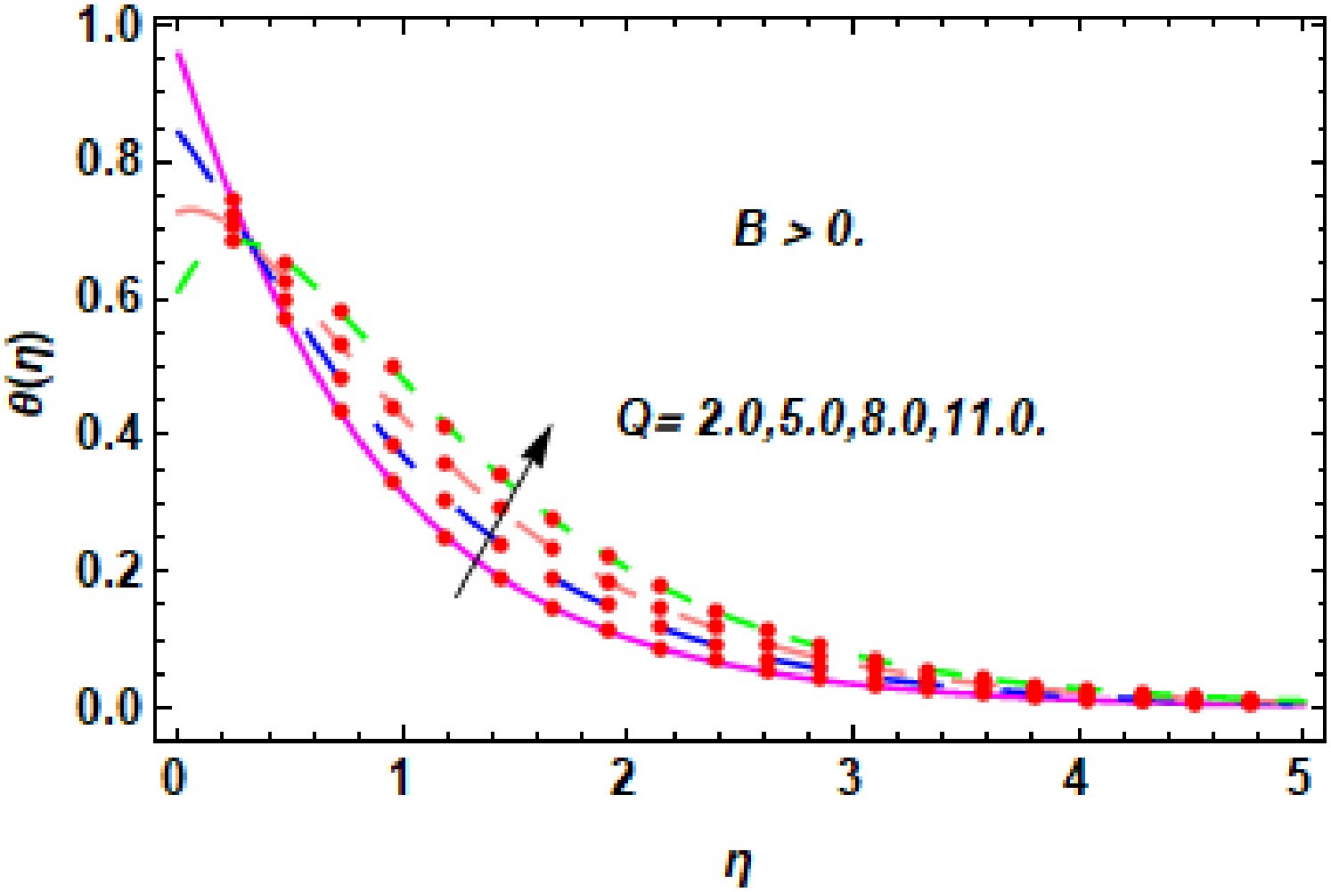
The nature of energy field for *Q*.

**Fig 23:**
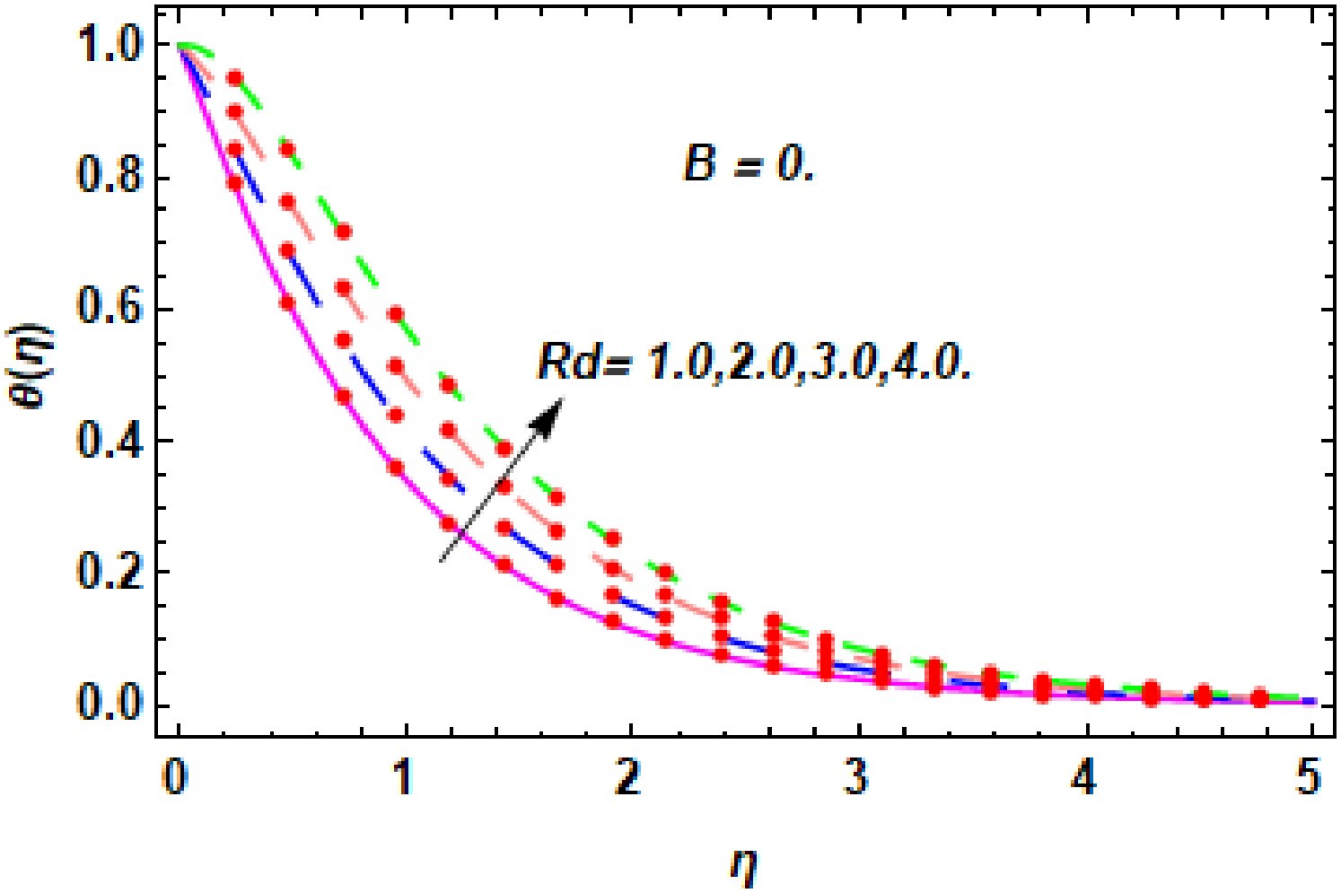
The nature of energy field for *Rd*.

For the description of the current study, the most powerful technique called the homotopy analysis technique is employed. The influence of emerging parameters on the hybrid nanofluid velocity and temperature are computed and debated in detail. Fig 1 displays the physical diagram of the flow problem. Fig 2 explains the convergence analysis of the homotopy solution. Figs 3–30 are drawn for the evaluation of hybrid nanofluid velocity and temperature for distinct parameters. Table 2, demonstrates the influence of Eckert number *Ec*, stretching parameter *λ*, suction parameter *S*, mixed convection parameter *λ*_1_, heat generation *Q*, magnetic field *M* and radiation parameter *Rd* on the skin fraction coefficient of the hybrid nanofluid for both slip *A* > 0 and no-slip *A* = 0 conditions. From Table 2, it is clear that the skin fraction $\sqrt{{\text{Re}}_{x}}C_{fx}$ of the hybrid nanofluid is enhanced with the increasing of *Ec*, *λ*, *λ*_1_, *Q* and *Rd* for no-slip condition *A* = 0. For slip condition *A* > 0, the hybrid nanofluid skin friction coefficient is increased via *λ*, *λ*_1_, *S* and *Rd*. Also, it is observed the higher estimation of *S* and *M* reduced the hybrid nanofluid skin friction coefficient $\sqrt{{\text{Re}}_{x}}C_{fx}$ for no-slip condition *A* = 0. *Ec*, *Q* and *M* diminution the hybrid nanofluid skin friction coefficient for slip condition *A* > 0. The outcomes of hybrid nanofluid Nusselt number $\frac{1}{\sqrt{{\text{Re}}_{x}}}Nu_{x}$ for no-slip *B* = 0 and slip *B* > 0 conditions due to varied values of Eckert number *Ec*, stretching parameter *λ*, suction parameter *S*, mixed convection parameter *λ*_1_, heat generation parameter *Q*, magnetic field parameter *M* and radiation parameter *Rd* are discussed in Table 3. Table 3 shows that the results of Nusselt number $\frac{1}{\sqrt{{\text{Re}}_{x}}}Nu_{x}$ are rises when the dimensionless parameter *Ec*, *λ*, *S*, *λ*_1_ and *Rd* are surges for no-slip condition *B* = 0, but the Nusselt number of hybrid nanofluid is condensed for *Ec*, *Q* and *M* for slip condition *B* > 0. The decrement in Nusselt number $\frac{1}{\sqrt{{\text{Re}}_{x}}}Nu_{x}$ for *M* and *Q* for no-slip condition *B* = 0 and $\frac{1}{\sqrt{{\text{Re}}_{x}}}Nu_{x}$ is increased for varying estimations of *λ*, *S*, *λ*_1_ and *Rd* for slip condition *B* > 0 is also examined in Table 3.

**Fig 24:**
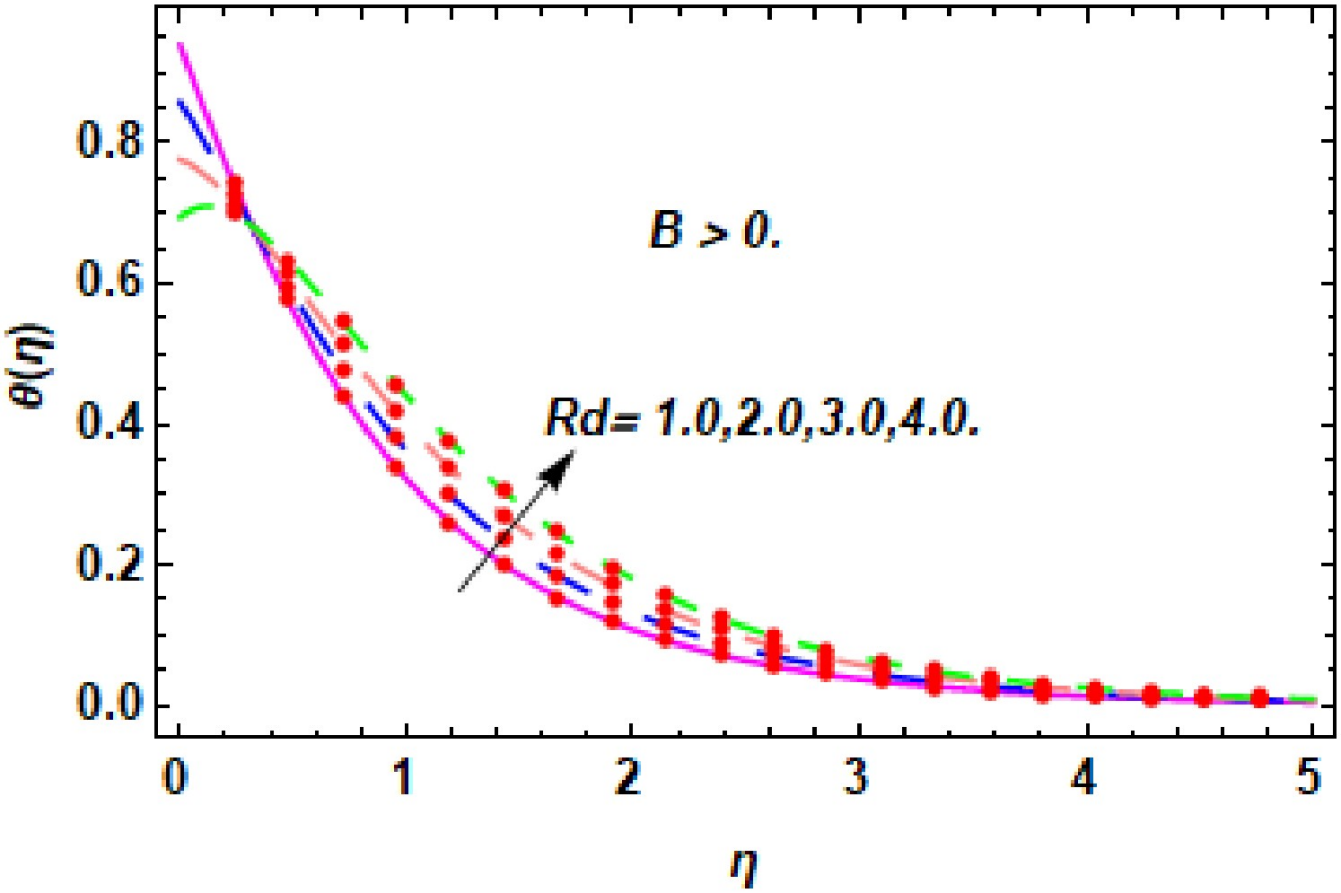
The nature of energy field for *Rd*.

**Fig 25:**
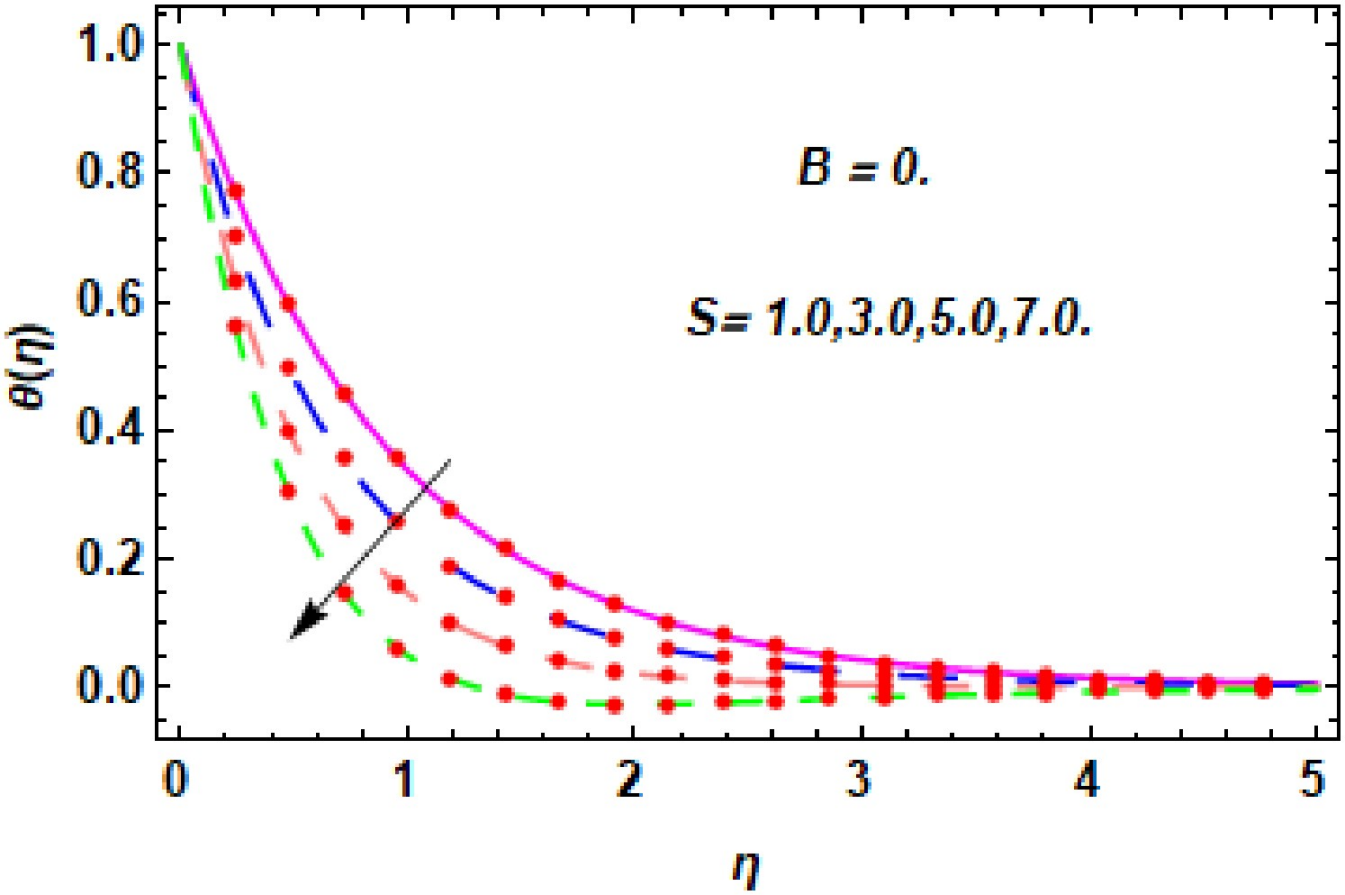
The nature of energy field for *S*.

**Fig 26:**
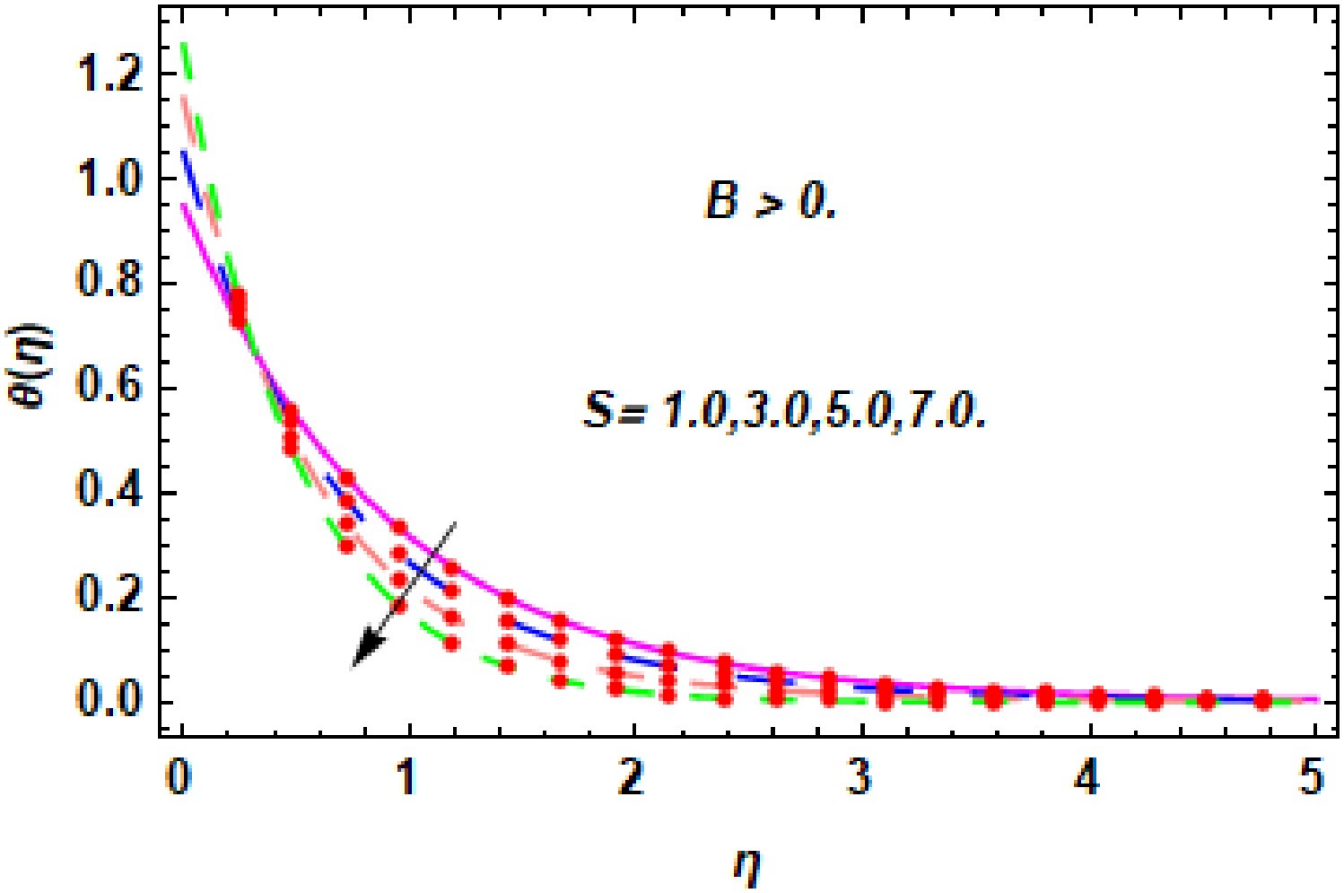
The nature of energy field for *S*.

**Fig 27:**
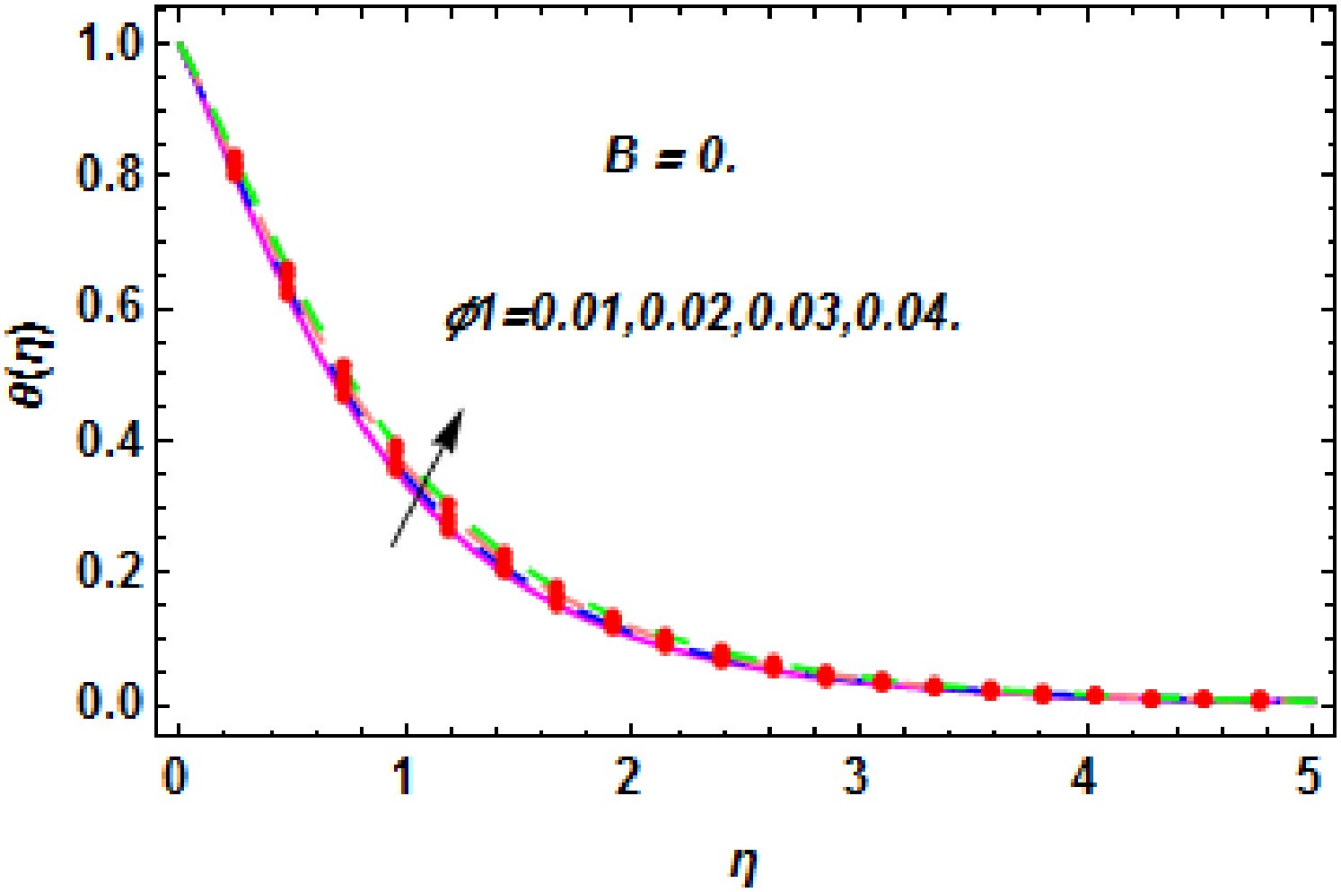
The nature of energy field for *ϕ*_1_.

**Fig 28:**
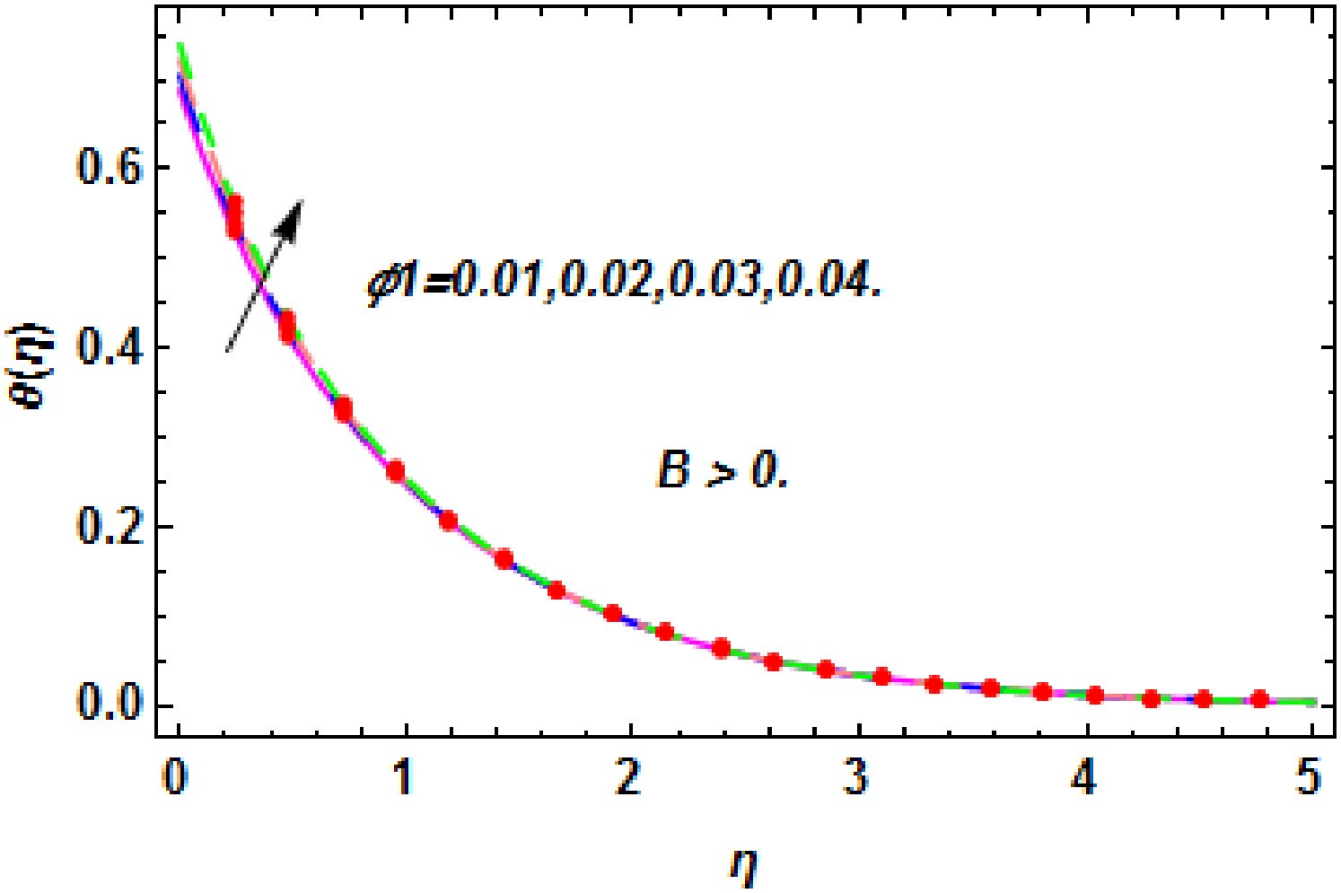
The nature of energy field for *ϕ*_1_.

**Fig 29:**
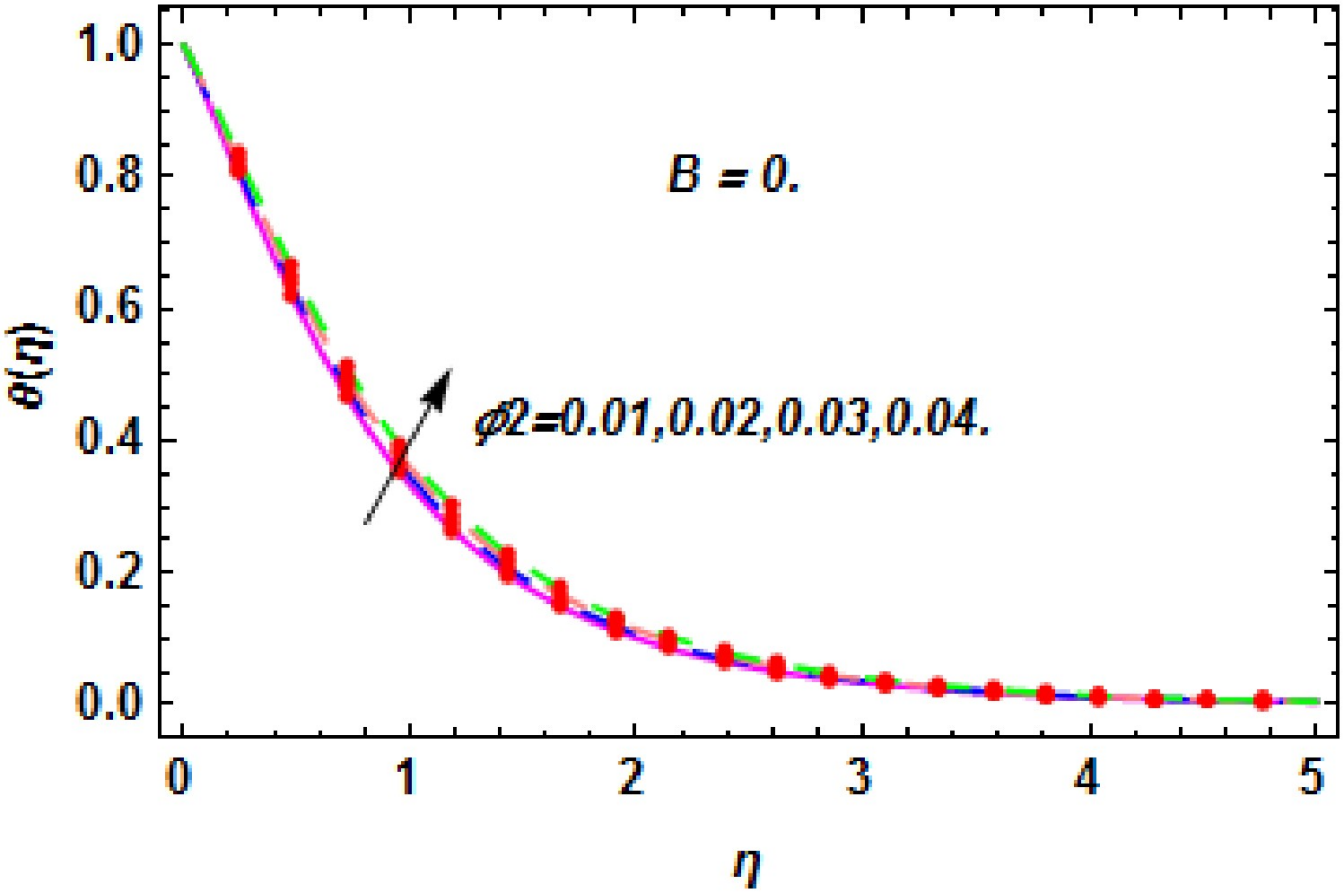
The nature of energy field for *ϕ*_2_.

**Fig 30:**
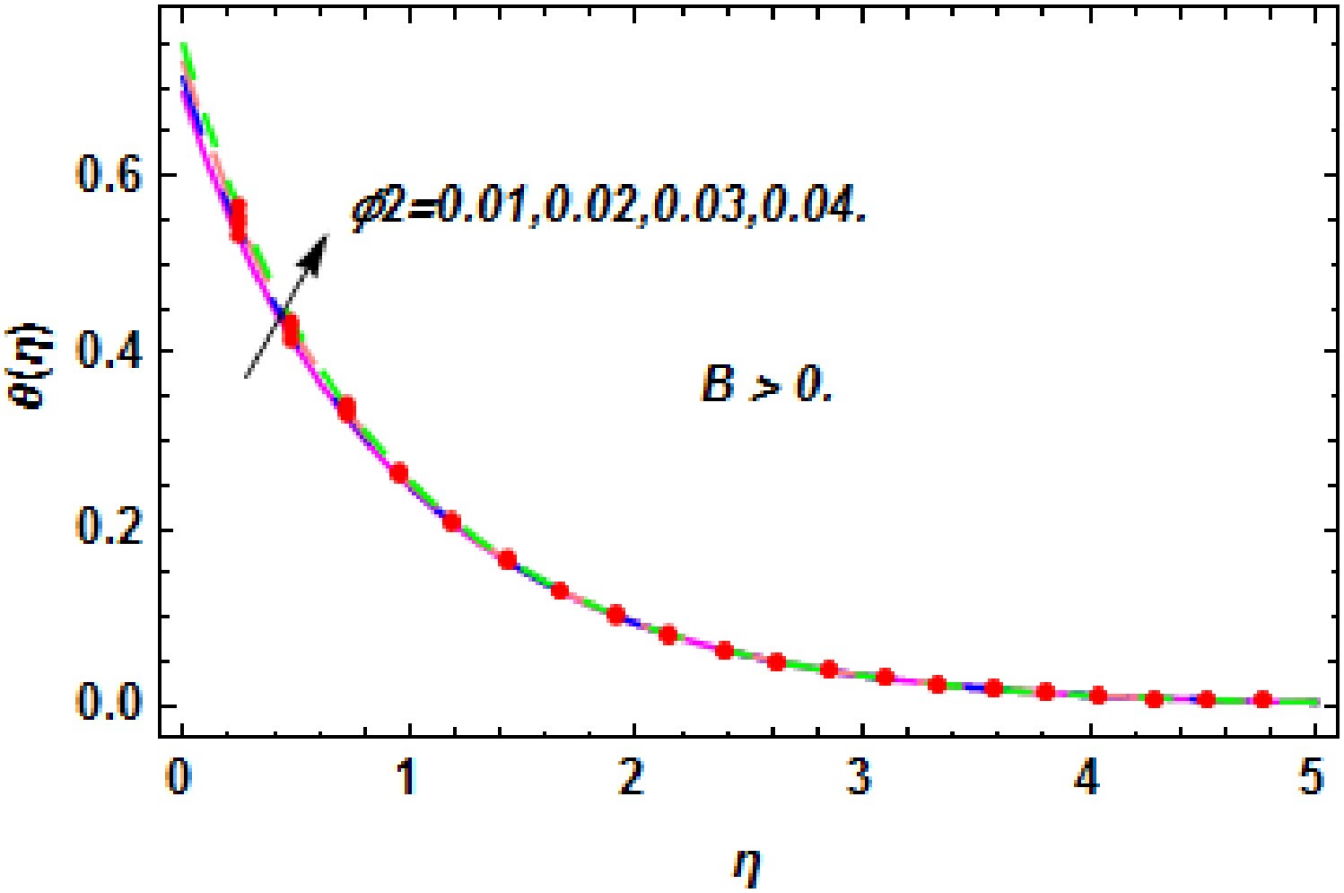
The nature of energy field for *ϕ*_2_.

**Table 2:** Influences of *Ec*, *λ*, *S*, *λ*_1_, *Q*, *M* and *Rd* on *C*_*fx*_, when *A* = 0and *A* > 0.

*Ec*	*λ*	*S*	*λ*_1_	*Q*	*M*	*Rd*	*C*_*fx*_ when *A* = 0	*C*_*fx*_ when *A* > 0
2.0							0.395895	0.363555
4.0							0.405062	0.363348
6.0							0.414229	0.363142
8.0							0.423396	0.362935
	1.0						0.570825	1.156689
	2.0						0.570826	0.032131
	3.0						0.745712	0.128522
	4.0						0.748393	0.289172
		0.1					0.231906	0.213501
		0.2					0.229906	0.215349
		0.3					0.215836	0.223595
		0.4					0.199624	0.231760
			1.0				0.519923	0.193570
			2.0				0.850618	0.316978
			3.0				1.181313	0.440386
			4.0				1.512008	0.563795
				1.0			0.205167	0.171709
				1.5			0.223415	0.168014
				2.0			0.241662	0.164320
				2.5			0.259909	0.160625
					1.2		0.636720	0.511270
					1.4		0.607520	0.487867
					1.6		0.578320	0.464507
					1.8		0.549120	0.441147
						0.3	0.387645	0.144206
						0.6	0.404007	0.152387
						0.9	0.420368	0.160568
						1.2	0.436730	0.168749

**Table 3:** Influence of *Ec*, *λ*, *S*, *λ*_1_, *Q*, *M* and *Rd* on *Nu*_*x*_, when *B* = 0 and *B* > 0.

*Ec*	*λ*	*S*	*λ*_1_	*Q*	*M*	*Rd*	*Nu*_*x*_ when *B* = 0	*Nu*_*x*_ when *B* > 0
2.0							1.912941	1.635948
4.0							2.037571	1.610227
6.0							2.162201	1.584506
8.0							2.286830	1.558784
	1.0						1.883593	1.740258
	2.0						1.883594	1.740259
	3.0						2.423480	4.568441
	4.0						2.748967	5.625370
		0.1					0.967897	0.332654
		0.2					0.967898	0.724576
		0.3					1.293757	1.083114
		0.4					1.697954	1.562308
			1.0				1.855277	0.122225
			2.0				1.991536	0.135907
			3.0				2.127794	0.250411
			4.0				2.264052	0.636729
				1.0			1.737117	0.944546
				1.5			1.340573	0.930309
				2.0			0.919420	0.597884
				2.5			0.473660	0.494101
					1.2		1.442347	0.415955
					1.4		1.439466	0.414941
					1.6		1.436586	0.413927
					1.8		1.433705	0.412913
						0.3	1.800774	0.676752
						0.6	2.114348	0.583368
						0.9	2.587347	0.777090
						1.2	3.355439	1.484031

### 5.1 Velocity profile

Figs 3–14 are displayed to see the variation in a hybrid nanofluid velocity profile via different values of stretching parameter *λ*, mixed convection parameter *λ*_1_, magnetic field parameter *M* and suction parameter *S* and nanoparticles concentrations *ϕ*_1_ and *ϕ*_2_ for both slip condition (*A* > 0) and no-slip (*A* = 0) conditions. From Fig 3, it is noted that the hybrid nanofluid velocity is increased when the dimensionless stretching parameter *λ* is enhanced for no-slip condition (*A* = 0). Fig 4 demonstrates that the velocity profile increments due to the increase of *λ* for slip condition (*A* > 0). The illustration of velocity profile against mixed convection parameter *λ*_1_ for no-slip condition (*A* = 0) is examined in Fig 5. From this figure, it is clear that the mixed convection parameter *λ*_1_ raised the hybrid nanofluid velocity. The change in hybrid nanofluid velocity profile for mixed convection parameter *λ*_1_ for slip condition (*A* > 0) is displayed in Fig 6. For growing values of *λ*_1_, the velocity profile is also increasing. The decrement in hybrid nanofluid velocity profile for varying values of *M* for no-slip condition (*A* = 0) is displayed in Fig 7. Higher values of magnetic field produced more and more frictional forces that have to decrease the fluid velocity. Physically, the heat transfer rate is enhanced due to the production of Lorentz forced by larger values of magnetic field parameter. Further, the heat transfer rate is increased due to the magnetic field parameter because there will be a large amount of energy is store in the fluid. Moreover, it is examined that the thermal and momentum boundary layer of the hybrid nanofluid becomes thinner with the increase of magnetic field parameter. Fig 8 depicted the behavior of hybrid nanofluid velocity profile for distinct estimation of magnetic field parameter *M* for slip condition (*A* > 0). The velocity profile is declined with the upsurge in *M*. It is only because the Lorentz force is generated by the implementation of the strong magnetic field in a fluid. This Lorentz force causes the resistance in the motion of the fluid particles that lowers the velocity of the fluid. Figs 9 and 10 are plotted to check the impact of *S* for no-slip (*A* = 0) and slip (*A* > 0) conditions on the velocity profile. In these graphs, the reduction in hybrid nanofluid velocity profile is obtained for rising estimation of *S* for both no-slip and slip conditions and the boundary layer thickness is weakened. Figs 11 and 12 portrayed the explanation of velocity profile for nanoparticles concentration of *Ag*(silver) for the no-slip (*A* = 0) and slip (*A* > 0) conditions. For increasing values of *ϕ*_1_, the decline in velocity profile is noted for both slip (*A* > 0) and no-slip (*A* = 0) conditions. The illustration of hybrid nanofluid velocity for both slip (*A* > 0) and the no-slip (*A* = 0) conditions via discrete estimation of *ϕ*_2_ are designed in Figs 13 and 14. When the nanoparticles concentration of *MgO*(Magnesium oxide) *ϕ*_2_ is large, then the hybrid nanofluid velocity is small in these figures for both no-slip and slip conditions. Physically, the weakening characteristic of the momentum boundary layer is caused by increasing values of nanoparticles volume fractions *ϕ*_1_ and *ϕ*_2_, therefore the velocity of the hybrid nanofluid is decreased.

### 5.2 Temperature profile

The consequence of Eckert number *Ec*, stretching parameter *λ*, magnetic field parameter *M*, heat generation parameter *Q*, radiation parameter *Rd*, suction parameter *S* and nanoparticles concentration *ϕ*_1_ and *ϕ*_2_ for the no-slip (*B* = 0) and slip (*B* > 0) conditions on the hybrid nanofluid temperature are presented in Figs 15–30. Fig 15 analyzed the effect of Eckert number over fluid temperature for the no-slip condition (*B* = 0). It is noted that the improvement in *Ec* boosted the hybrid nanofluid temperature. The performance of the Eckert number on the hybrid nanofluid temperature for slip condition (*B* > 0) is determined in Fig 16. It is distinguished that with the augmentation of Eckert number, the temperature of the hybrid nanofluid is amplified. The process of conversion of mechanical energy into thermal energy becomes faster due to the increase of Eckert number that leads to enlarged the fluid temperature. The Eckert number is a term that describes the relationship between the flow of kinetic energy and heat enthalpy variation. It means that with the increase of Eckert, the kinetic energy of the hybrid nanofluid is amplified. Furthermore, the temperature is commonly defined as the average kinetic energy. That’s why the temperature of the hybrid nanofluid is enhanced with the enhancement of Eckert number. Fig 17 is sketched for the evaluation of temperature profile against stretching parameter *λ* for no-slip condition (*B* = 0). It is found that when the values of *λ* is increased, then the fluid temperature is decayed. The enhancement in hybrid nanofluid temperature is examined for higher values of stretching parameter *λ* for the slip condition (*B* > 0) in Fig 18. For the case of no-slip condition (*B* = 0), the effect of the *M* on the hybrid nanofluid temperature is disclosed in Fig 19. The augmentation in fluid temperature is detected for fluctuating values of magnetic field parameter *M*. Fig 20 is drawn to evaluate the effect of the magnetic field parameter for slip condition (*B* > 0) on the hybrid nanofluid temperature profile. From this figure, it is noticed that the temperature profile is boosted when the magnetic field parameter is upgraded. As it is detected from the above-mentioned velocity profile that fluid motion is decreased due to an increase of magnetic field parameter. Because of the slow motion of the fluid, the kinetic energy is transformed into heat energy, causing the temperature to rise. Moreover, it is examined that the thermal and momentum boundary layer of the hybrid nanofluid becomes thinner with the enhancement of the magnetic field. The inverse relation between the density of the hybrid nanofluid and magnetic parameter is observed. As a result, increasing the magnetic field parameter causes the fluid density to decrease, resulting in temperature increases. The influence of fluid temperature profile for different values of heat generation parameter *Q* for no-slip condition (*B* = 0) is inspected in Fig 21). It shows that the temperature profile increases as the value of heat generation *Q* augments. The role of *Q* on the temperature of the fluid for slip condition (*B* > 0) is studied in Fig 22. It is comprehended that the estimation in heat generation parameter *Q* leads to the escalation in temperature of the hybrid nanofluid. Fig 23 is used to detect the effect of radiation parameter *Rd* for no-slip condition (*B* = 0) on fluid temperature profile which explained that temperature profile is enhanced for larger values of *Rd*. The enrichment in boundary layer thickness is occur due to the rise of fluid temperature. An increment in radiation parameter provides an additional amount of heat to hybrid nanofluid and as a result the fluid temperature heightens. Also, the thermal diffusivity of the hybrid nanofluid is enhanced for higher values of radiation parameter, that’s why the hybrid nanofluid temperature is increased. Fig 24 portrays the influence of radiation parameter for slip condition (*B* > 0) on the fluid temperature profile. The expansion in temperature is exhibited for intensifying values of radiation parameter. In Fig 25, for the no-slip condition (*B* = 0), the disparity of temperature profile for increasing values of *S* is discussed. The outcome from this figure shows that decrement in hybrid nanofluid temperature profile is perceived for varied values of *S*. Fig 26 presents the variation in temperature profile against *S* for the case of slip condition (*B* > 0). It revealed that for rising values of *S*, the fluid temperature profile is reduced which results to decaying conduct in the boundary layer. Physically, a considerable amount of fluid flows outside the walls of the sheet during the process of suction which slows down the motion and boundary layer thickness of the fluid, that’s why the fluid temperature is diminished. Figs 27 and 28 are displayed to describe the influence of nanoparticles concentration *ϕ*_1_ on the hybrid nanofluid temperature for the slip (*B* > 0) and no-slip (*B* = 0) conditions. For both no-slip and slip conditions, the hybrid nanofluid temperature is increased with the rise of nanoparticles concentration (*ϕ*_1_) of *Ag*(silver). Figs 29 and 30 shows the disparity of hybrid nanofluid temperature profile for serval estimation of nanoparticles concentration (*ϕ*_2_) of *MgO*(Magnesium oxide). From this analysis, it is observed that with the augmentation of nanoparticles concentration *ϕ*_2_, the temperature of hybrid nanofluid is heightened. By adding the nanoparticles to base fluids improves thermal conductivity and so improves the thermal efficiency of the hybrid nanofluid. That’s why the hybrid nanofluid temperature is increased against growing values of nanoparticles volume fraction.

## 6. Conclusion

This article aims to investigate the MHD flow of hybrid nanofluid toward the stretching surface in the presence of Joule heating, thermal radiation and heat source/sink effects. The present model consists of momentum and temperature equations along with slip conditions. The fluid motion is generated due to the stretching of the surface. The homotopy analysis method (HAM) is employed for the discretization of the current study. The main finding of the present study is

- The skin friction coefficient $\sqrt{{\text{Re}}_{x}}C_{fx}$ of the hybrid nanofluid is increased with the increase in Eckert number *Ec*, stretching parameter *λ*, heat generation parameter *Q* and radiation parameter *Rd* for both no-slip (*A* = 0) and slip (*A* > 0) conditions.
- The growth in suction and magnetic field parameters lead to a decline the skin friction coefficient $\sqrt{{\text{Re}}_{x}}C_{fx}$ of the hybrid nanofluid for both no-slip (*A* = 0) and slip (*A* > 0) conditions.
- For the case of slip (*B* > 0) and no-slip (*B* = 0) conditions, the improvement in Eckert number *Ec*, stretching parameter *λ* suction parameter *S*, mixed convection parameter *λ*_1_, heat generation *Q* and radiation parameter *Rd* have augmented the Nusselt number $\frac{1}{\sqrt{{\text{Re}}_{x}}}Nu_{x}$.
- The declining conduct in $\frac{1}{\sqrt{{\text{Re}}_{x}}}Nu_{x}$ is detected for both no-slip (*B* = 0) and slip (*B* > 0) conditions against rising values of magnetic field parameter.
- The upward trend is observed in hybrid nanofluid velocity for both stretching *λ* and mixed convection *λ*_1_ parameters for both no-slip (*A* = 0) and slip (*A* > 0) conditions.
- For both the cases of no-slip (*A* = 0) and slip (*A* > 0) conditions, the increment in magnetic field parameter *M*, volume fraction of *Ag*(silver) and volume fraction of *MgO*(Magnesium oxide) have decreased the hybrid nanofluid velocity.
- When the Eckert number *Ec*, stretching parameter *λ*, magnetic field parameter *M*, heat generation parameter *Q*, radiation parameter *Rd*, the volume fraction of *Ag*(silver) and volume fraction of *MgO*(Magnesium oxide) are augmented, then the temperature of the hybrid nanofluid is also enhanced for both no-slip (*B* = 0) and slip (*B* > 0) conditions.
- The temperature of the hybrid nanofluid is declined for increasing values of the suction parameter *S* for both no-slip (*B* = 0) and slip (*B* > 0) conditions.

## Supporting information

S1 Abbreviations (DOCX)

## References

[1] Shoaib M., Raja M. A. Z., Sabir M. T., Islam S., Shah Z., Kumam P., et al. (2020). Numerical investigation for rotating flow of MHD hybrid nanofluid with thermal radiation over a stretching sheet. *Scientific Reports*, 10(1), 1–15. 33116167 10.1038/s41598-020-75254-8 PMC7595176

[2] Al-Hanaya A. M., Sajid F., Abbas N., & Nadeem S. (2020). Effect of SWCNT and MWCNT on the flow of micropolar hybrid nanofluid over a curved stretching surface with induced magnetic field. *Scientific Reports*, 10(1), 1–18. 32444647 10.1038/s41598-020-65278-5 PMC7244560

[3] Lund L. A., Omar Z., Khan I., & Sherif E. S. M. (2020). Dual solutions and stability analysis of a hybrid nanofluid over a stretching/shrinking sheet executing MHD flow. *Symmetry*, 12(2), 276.

[4] Shoaib M., Raja M. A. Z., Sabir M. T., Awais M., Islam S., Shah Z., et al. (2021). Numerical analysis of 3-D MHD hybrid nanofluid over a rotational disk in presence of thermal radiation with Joule heating and viscous dissipation effects using Lobatto IIIA technique. *Alexandria Engineering Journal*, 60(4), 3605–3619.

[5] Ahmad F., Abdal S., Ayed H., Hussain S., Salim S., & Almatroud A. O. (2021). The improved thermal efficiency of Maxwell hybrid nanofluid comprising of graphene oxide plus silver/kerosene oil over stretching sheet. *Case Studies in Thermal Engineering*, 27, 101257.

[6] Alhussain Z. A., & Tassaddiq A. (2021). Thin Film Blood Based Casson Hybrid Nanofluid Flow with Variable Viscosity. *Arabian Journal for Science and Engineering*, 1–8.

[7] Sreedevi P., & Reddy P. S. (2021). Williamson hybrid nanofluid flow over swirling cylinder with Cattaneo–Christov heat flux and gyrotactic microorganism. *Waves in Random and Complex Media*, 1–28.

[8] Roy N. C., & Pop I. (2020). Flow and heat transfer of a second-grade hybrid nanofluid over a permeable stretching/shrinking sheet. *The European Physical Journal Plus*, 135(9), 1–19.

[9] Tlili I., Mustafa M. T., Kumar K. A., & Sandeep N. (2020). Effect of asymmetrical heat rise/fall on the film flow of magnetohydrodynamic hybrid ferrofluid. *Scientific reports*, 10(1), 1–11. 32317721 10.1038/s41598-020-63708-y PMC7174402

[10] Kempannagari A. K., Buruju R. R., Naramgari S., & Vangala S. (2020). Effect of Joule heating on MHD non-Newtonian fluid flow past an exponentially stretching curved surface. *Heat Transfer*, 49(6), 3575–3592.

[11] Kumar K. A., Reddy J. R., Sugunamma V., & Sandeep N. (2018). Magnetohydrodynamic Cattaneo-Christov flow past a cone and a wedge with variable heat source/sink. *Alexandria engineering journal*, 57(1), 435–443.

[12] Venkata Ramudu A. C., Anantha Kumar K., Sugunamma V., & Sandeep N. (2020). Heat and mass transfer in MHD Casson nanofluid flow past a stretching sheet with thermophoresis and Brownian motion. *Heat Transfer*, 49(8), 5020–5037.

[13] Kumar K. A., Sugunamma V., Sandeep N., & Mustafa M. T. (2019). Simultaneous solutions for first order and second order slips on micropolar fluid flow across a convective surface in the presence of Lorentz force and variable heat source/sink. *Scientific reports*, 9(1), 1–14. 31604996 10.1038/s41598-019-51242-5 PMC6788988

[14] Ramadevi B., Kumar K. A., Sugunamma V., & Sandeep N. (2019). Influence of non-uniform heat source/sink on the three-dimensional magnetohydrodynamic Carreau fluid flow past a stretching surface with modified Fourier’s law. *Pramana*, 93(6), 1–11.

[15] Kumar K. A., Sugunamma V., & Sandeep N. (2020). Influence of viscous dissipation on MHD flow of micropolar fluid over a slendering stretching surface with modified heat flux model. *Journal of Thermal Analysis and Calorimetry*, 139(6), 3661–3674.

[16] Idowu A. S., & Falodun B. O. (2020). Effects of thermophoresis, Soret-Dufour on heat and mass transfer flow of magnetohydrodynamics non-Newtonian nanofluid over an inclined plate. *Arab Journal of Basic and Applied Sciences*, 27(1), 149–165.

[17] Islam S., Khan A., Deebani W., Bonyah E., Alreshidi N. A., & Shah Z. (2020). Influences of Hall current and radiation on MHD micropolar non-Newtonian hybrid nanofluid flow between two surfaces. *AIP Advances*, 10(5), 055015.

[18] Jain S., & Bhargava R. (2020). Numerical simulation of free convection of MHD non-Newtonian nanofluid within a square wavy enclosure using Meshfree method. *International Journal for Computational Methods in Engineering Science and Mechanics*, 22(1), 32–44.

[19] El-Dabe N. T., Abou-Zeid M. Y., Mohamed M. A., & Abd-Elmoneim M. M. (2021). MHD peristaltic flow of non-Newtonian power-law nanofluid through a non-Darcy porous medium inside a non-uniform inclined channel. *Archive of Applied Mechanics*, 91(3), 1067–1077.

[20] Kotha G., Kolipaula V. R., Rao M. V. S., Penki S., & Chamkha A. J. (2020). Internal heat generation on bioconvection of an MHD nanofluid flow due to gyrotactic microorganisms. *The European Physical Journal Plus*, 135(7), 1–19.

[21] Waqas H., Imran M., Hussain S., Ahmad F., Khan I., Nisar K. S., et al. (2020). Numerical simulation for bioconvection effects on MHD flow of Oldroyd-B nanofluids in a rotating frame stretching horizontally. *Mathematics and Computers in Simulation*, 178, 166–182.

[22] Aziz A., & Shams M. (2020). Entropy generation in MHD Maxwell nanofluid flow with variable thermal conductivity, thermal radiation, slip conditions, and heat source. *AIP Advances*, 10(1), 015038.

[23] Gupta S., Kumar D., & Singh J. (2020). Analytical study for MHD flow of Williamson nanofluid with the effects of variable thickness, nonlinear thermal radiation and improved Fourier’s and Fick’s Laws. *SN Applied Sciences*, 2(3), 1–12.

[24] Ashwinkumar G. P., Samrat S. P., & Sandeep N. (2021). Convective heat transfer in MHD hybrid nanofluid flow over two different geometries. *International Communications in Heat and Mass Transfer*, 127, 105563.

[25] Samrat S. P., Ashwinkumar G. P., & Sandeep N. (2021). Simultaneous solutions for convective heat transfer in dusty-nano-and dusty-hybrid nanoliquids. *Proceedings of the Institution of Mechanical Engineers*, *Part E*: *Journal of Process Mechanical Engineering*, 09544089211043605.

[26] Chalavadi S., Madde P., Naramgari S., & Gangadhar Poojari A. Effect of variable heat generation/absorption on magnetohydrodynamic Sakiadis flow of Casson/Carreau hybrid nanoliquid due to a persistently moving needle. *Heat Transfer*.

[27] Ashwinkumar G. P. (2021). Heat and mass transfer analysis in unsteady MHD flow of aluminum alloy/silver-water nanoliquid due to an elongated surface. *Heat Transfer*, 50(2), 1679–1696.

[28] Mabood F., Ashwinkumar G. P., & Sandeep N. (2021). Simultaneous results for unsteady flow of MHD hybrid nanoliquid above a flat/slendering surface. *Journal of Thermal Analysis and Calorimetry*, 146(1), 227–239.

[29] Tlili I., Nabwey H. A., Ashwinkumar G. P., & Sandeep N. (2020). 3-D magnetohydrodynamic AA7072-AA7075/methanol hybrid nanofluid flow above an uneven thickness surface with slip effect. *Scientific reports*, 10(1), 1–13. 32144369 10.1038/s41598-020-61215-8 PMC7060228

[30] Tlili I., Nabwey H. A., Ashwinkumar G. P., & Sandeep N. (2020). 3-D magnetohydrodynamic AA7072-AA7075/methanol hybrid nanofluid flow above an uneven thickness surface with slip effect. *Scientific reports*, 10(1), 1–13. 32144369 10.1038/s41598-020-61215-8 PMC7060228

[31] Akbari O. A., Toghraie D., Karimipour A., Marzban A., & Ahmadi G. R. (2017). The effect of velocity and dimension of solid nanoparticles on heat transfer in non-Newtonian nanofluid. *Physica E*: *Low-Dimensional Systems and Nanostructures*, 86, 68–75.

[32] Xiong Q., Bozorg M. V., Doranehgard M. H., Hong K., & Lorenzini G. (2020). A CFD investigation of the effect of non-Newtonian behavior of Cu–water nanofluids on their heat transfer and flow friction characteristics. *Journal of Thermal Analysis and Calorimetry*, 139(4), 2601–2621.

[33] Mahabaleshwar U. S., Nagaraju K. R., Kumar P. V., Nadagouda M. N., Bennacer R., & Sheremet M. A. (2020). Effects of Dufour and Soret mechanisms on MHD mixed convective-radiative non-Newtonian liquid flow and heat transfer over a porous sheet. *Thermal Science and Engineering Progress*, 16, 100459.

[34] Mukhtar T., Jamshed W., Aziz A., & Al-Kouz W. (2020). Computational investigation of heat transfer in a flow subjected to magnetohydrodynamic of Maxwell nanofluid over a stretched flat sheet with thermal radiation. *Numerical Methods for Partial Differential Equations*.

[35] Aamir H., & Khan M. (2020). Heat and mass transport phenomena of nanoparticles on time-dependent flow of Williamson fluid towards heated surface. *Neural Computing & Applications*, 32(8), 3253–3263.

[36] Hayat T., Ullah H., Ahmad B., & Alhodaly M. S. (2021). Heat transfer analysis in convective flow of Jeffrey nanofluid by vertical stretchable cylinder. *International Communications in Heat and Mass Transfer*, 120, 104965.

[37] Khan M. I., Alzahrani F., & Hobiny A. (2020). Heat transport and nonlinear mixed convective nanomaterial slip flow of Walter-B fluid containing gyrotactic microorganisms. *Alexandria Engineering Journal*, 59(3), 1761–1769.

[38] Waini I., Ishak A., & Pop I. (2020). MHD flow and heat transfer of a hybrid nanofluid past a permeable stretching/shrinking wedge. *Applied Mathematics and Mechanics*, 41(3), 507–520.

[39] Alaidrous A. A., & Eid M. R. (2020). 3-D electromagnetic radiative non-Newtonian nanofluid flow with Joule heating and higher-order reactions in porous materials. *Scientific Reports*, 10(1), 1–19. 32884033 10.1038/s41598-020-71543-4 PMC7471956

[40] Hafeez A., Khan M., Ahmed A., & Ahmed J. (2021). Features of Cattaneo-Christov double diffusion theory on the flow of non-Newtonian Oldroyd-B nanofluid with Joule heating. *Applied Nanoscience*, 1–8.

[41] Khan A., Shah Z., Alzahrani E., & Islam S. (2020). Entropy generation and thermal analysis for rotary motion of hydromagnetic Casson nanofluid past a rotating cylinder with Joule heating effect. *International Communications in Heat and Mass Transfer*, 119, 104979.

[42] Kumar A., Tripathi R., Singh R., & Chaurasiya V. K. (2020). Simultaneous effects of nonlinear thermal radiation and Joule heating on the flow of Williamson nanofluid with entropy generation. *Physica A*: *Statistical Mechanics and its Applications*, 551, 123972.

[43] Khashi’ie N. S., Arifin N. M., Pop I., & Wahid N. S. (2020). Flow and heat transfer of hybrid nanofluid over a permeable shrinking cylinder with Joule heating: a comparative analysis. *Alexandria Engineering Journal*, 59(3), 1787–1798.

[44] Uddin I., Ullah I., Ali R., Khan I., & Nisar K. S. (2021). Numerical analysis of nonlinear mixed convective MHD chemically reacting flow of Prandtl–Eyring nanofluids in the presence of activation energy and Joule heating. *Journal of Thermal Analysis and Calorimetry*, 145(2), 495–505.

[45] Kazemi M. A., Javanmard M., Taheri M. H., & Askari N. (2020). Heat transfer investigation of the fourth-grade non-Newtonian MHD fluid flow in a plane duct considering the viscous dissipation, joule heating and forced convection on the walls. *SN Applied Sciences*, 2(10), 1–14.

[46] Ur Rasheed H., Saleem S., Islam S., Khan Z., Khan W., Firdous H., et al. (2021). Effects of Joule heating and viscous dissipation on magnetohydrodynamic boundary layer flow of Jeffrey nanofluid over a vertically stretching cylinder. *Coatings*, 11(3), 353.

[47] Abu Bakar S., Md Arifin N., Khashi’ie N. S., & Bachok N. (2021). Hybrid Nanofluid Flow over a Permeable Shrinking Sheet Embedded in a Porous Medium with Radiation and Slip Impacts. *Mathematics*, 9(8), 878.

[48] Wahid N. S., Arifin N. M., Khashi’ie N. S., & Pop I. (2021). Hybrid nanofluid slip flow over an exponentially stretching/shrinking permeable sheet with heat generation. *Mathematics*, 9(1), 30.

[49] Yashkun U., Zaimi K., Ishak A., Pop I., & Sidaoui R. (2020). Hybrid nanofluid flow through an exponentially stretching/shrinking sheet with mixed convection and Joule heating. *International Journal of Numerical Methods for Heat & Fluid Flow*.

[50] Ma Y., Mohebbi R., Rashidi M. M., & Yang Z. (2019). MHD convective heat transfer of Ag-MgO/water hybrid nanofluid in a channel with active heaters and coolers. *International Journal of Heat and Mass Transfer*, 137, 714–726.

[51] Zainal N. A., Nazar R., Naganthran K., & Pop I. (2021). Viscous dissipation and MHD hybrid nanofluid flow towards an exponentially stretching/shrinking surface. *Neural Computing and Applications*, 1–11.

[52] Liao S. (2012). *Homotopy analysis method in nonlinear differential equations* (pp. 153–165). Beijing: Higher education press.

