# Supplementary material for: Heat transfer analysis of the mixed convective flow of magnetohydrodynamic hybrid nanofluid past a stretching sheet with velocity and thermal slip conditions
Source: PLoS One. 2021 Dec 14;16(12):e0260854. doi: 10.1371/journal.pone.0260854 (PMC8670717; doi:10.1371/journal.pone.0260854)
Supplement: S1 Abbreviations — (DOCX) [file pone.0260854.s001.docx]

**Abbreviations**

| **Symbol** | **Name** | **Unit** |
| --- | --- | --- |
| $a$ | Positive constant |  |
| $A$, $B$ | Velocity slip parameter |  |
| $A_{1}$, $B_{1}$ | Velocity and thermal slip constants |  |
| $B_{0}$ | Applied magnetic field | $Kg/s^{2}A$ |
|  | Skin friction Coefficient |  |
| $f$ | Velocity profile |  |
| $g$ | Acceleration due gravity | $m/s^{2}$ |
| $k$ | Thermal conductivity | $W/mK$ |
| $k^{*}$ | Mean absorption coefficient |  |
| $L$ | Reference length | $m$ |
| $M$ | Magnetic field |  |
| $Nu_{x}$ | Nusselt number |  |
| $\Pr$ | Prandtl number |  |
| $q_{r}$ | Radiative heat flux | $W/m^{2}$ |
| $Q_{0}$ | Heat sink/source |  |
| $Q$ | Heat generation parameter |  |
| $Rd$ | Radiation parameter |  |
| $S$ | Suction/injection parameter |  |
| $T$ | Temperature | $K$ |
| $u_{w}$ | Stretching velocity | $m/s$ |
| $u,v$ | Velocity components | $m/s$ |
| **Greek letters** |  |  |
| $\theta$ | Temperature profile |  |
|  | Concentration profile |  |
|  | Volume fraction of $Ag$ |  |
|  | Volume fraction of $MgO$ |  |
| $\nu$ | kinematics viscosity | $m^{2}/s$ |
| $\sigma$ | Electrical conductivity | $S/m$ |
| $C_{p}$ | Heat capacitance | $J/KgK$ |
| $\rho$ | Density | $kg/m^{3}$ |
| $\sigma^{*}$ | Stefan-Boltzmann constant |  |
| $\mu$ | Dynamic viscosity | $kg/ms$ |
| $\psi$ | Stream function |  |
| $\beta_{T}$ | Thermal expansion coefficient | $1/K$ |
| $\lambda$ | Stretching parameter |  |
| $\lambda_{1}$ | Mixed convection parameter |  |
| **Subscripts** |  |  |
| $f$ | Fluid |  |
| $hnf$ | Hybrid nanofluid |  |
| $w$ | At the surface |  |
| $\infty$ | Free stream |  |
| $s_{1}$ | First nanoparticle |  |
| $s_{2}$ | Second nanoparticle |  |
